# Brain activation during social cognition predicts everyday perspective-taking: A combined fMRI and ecological momentary assessment study of the social brain

**DOI:** 10.1016/j.neuroimage.2020.117624

**Published:** 2020-12-17

**Authors:** Malin K. Hildebrandt, Emanuel Jauk, Konrad Lehmann, Lara Maliske, Philipp Kanske

**Affiliations:** a Institute of Clinical Psychology and Psychotherapy, Department of Psychology, Technische Universität Dresden, Dresden, Germany; b Department of Psychology, University of Graz, Graz, Austria; c Max Planck Institute for Human Cognitive and Brain Sciences, Stephanstraße 1A, 04103 Leipzig, Germany

**Keywords:** Empathy, Theory of mind, Mentalizing, fMRI, Ecological momentary assessment, Ecological validity

## Abstract

Identifying distinct neural networks underlying social affect (empathy, compassion) and social cognition (Theory of Mind) has advanced our understanding of social interactions. However, little is known about the relation of activation in these networks to psychological experience in daily life. This study (*N* = 122) examined the ecological validity of neural activation patterns induced by a laboratory paradigm of social affect and cognition with respect to social interactions in everyday life. We used the EmpaToM task, a naturalistic video-based paradigm for the assessment of empathy, compassion, and Theory of Mind, and combined it with a subsequent 14-day ecological momentary assessment protocol on social interactions. Everyday social affect was predicted by social affect experienced during the EmpaToM task, but not by related neural activation in regions of interest from the social affect network. In contrast, everyday social cognition was predicted by neural activation differences in the medial prefrontal cortex – a region of interest from the social cognition network – but not by social cognition performance in the EmpaToM task. The relationship between medial prefrontal cortex activation and everyday social cognition was stronger for spontaneous rather than deliberate perspective taking during the EmpaToM task, pointing to a distinction between propensity and capacity in social cognition. Finally, this neural indicator of Theory of Mind explained variance in everyday social cognition to a similar extent as an established selfreport scale. Taken together, this study provides evidence for the ecological validity of lab-based social affect and cognition paradigms when considering relevant moderating factors.

## 1 Introduction

Understanding others is a complex process that requires knowledge about others’ emotions, thoughts, beliefs and intentions. Unless explicitly expressed, these mental states are not directly accessible and the observer needs to ascertain them in an ongoing process. Psychological science has long examined whether the process of understanding others is subdivided into an affective and a cognitive component (Adolphs, 2008; Frith and Frith, 2010; Happé et al., 2017; Singer, 2006; Stietz et al., 2019). The affective component, termed *empathy*, describes affect sharing while being aware that one’s emotion originated in someone else, for example experiencing sadness oneself when comforting a friend (de Vignemont and Singer 2006). The cognitive component, *Theory of Mind* (*ToM*), describes cognitive inference of others’ mental states, building on what they say, how they act, and also on theories about their intentions and personalities (Frith and Frith, 2005). This skill requires the awareness that others’ mental states may differ from one’s own (Stuss et al., 2001). For example, one may hypothezise that a new colleague is shy based on their behavior and that they might therefore be grateful to be shown around without asking for it.

### 1.1 The neuroscience of social affect and cognition

Neuroscience methods, especially functional magnetic resonance imaging (fMRI), have greatly benefited the question whether the process of understanding others is subdivided into an affective and a cognitive component. By identifying distinct neural networks associated with affect sharing (empathizing/empathy), and inference of others’ mental and affective states (ToM), social neuroscience has provided support for the existence of these two separate paths to understanding others (Kanske, Böckler, et al., 2015; Kanske et al., 2016; Schurz et al., 2020; Singer, 2006; Stietz et al., 2019 Zaki and Ochsner, 2012). Regarding the affective component, meta-analyses identified the anterior insula (AI) and the anterior midcingulate cortex (aMCC) extending into the dorsomedial prefrontal cortex (dmPFC) / anterior cingulate cortex as neural regions reliably activated when observing others’ suffering (empathy for pain) - and when suffering oneself (Fan et al., 2011). Activation in these regions often covaries with negative affect (Lamm et al., 2011). This underlines shared activation or neural mirroring as the suspected mechanism underlying empathy – embodied sharing of others’ affective states. While empathy itself merely refers to affect sharing, it can be accompanied by compassion, also termed empathic concern, meaning a feeling of warmth and care directed towards others that may entail motivational aspects (e.g., to ease the other’s pain; Singer and Klimecki, 2014). As such, compassion needs to be differentiated from empathic affect-sharing, but both empathy and compassion are considered social affective processes (Kanske, 2018; Kanske et al., 2017; Preckel et al., 2018; Stietz et al., 2019). As opposed to empathy for pain, compassion induces activation in regions associated with positive emotions such as the ventral striatum (VS; Kanske, Böckler, et al., 2015; Klimecki et al., 2013).

Regarding the cognitive component, core regions associated with social cognition / ToM include the bilateral temporoparietal junction (TPJ) and temporal poles (TP), medial prefrontal cortex (mPFC), and precuneus/posterior cingulate cortex (PCC; Bzdok et al., 2012; Schurz et al., 2014). In contrast to embodied affect sharing, these regions are activated when reasoning about others’ mental and affective states. By identifying these different neural networks, neuroscience has advanced our understanding of social affect and cognition.

### 1.2 Ecological validity of social affect and cognition neuroimaging research

One aim of neuroscience is to gain further insight into how the human mind functions in everyday life. Yet, little is known about the link between neural activation and psychological experience outside the lab (Eisenberger et al., 2007; Hogenelst et al., 2015). This is due to the local constraints of neuroscience methodology, especially fMRI, confining its use to the laboratory. Typically, psychological processes studied by means of neuroscience methods are experimentally induced and then related brain activation is captured. Hence, the construct of interest is assessed only via psychological and neural processes in the laboratory and not as part of ongoing daily experience. High ecological validity is achieved when a measurement closely represents the usual conditions under which the process of interest occurs (Hogenelst et al., 2015). The first step towards improving ecological validity is the development of naturalistic paradigms. Social neuroscience has pursued this goal by developing more and more realistic approaches, for example video-based paradigms (Dziobek et al., 2006; Kanske, Böckler, et al., 2015), and continues to do so in the so-called second-person neuroscience approach (Lehmann et al., 2019; Schilbach et al., 2013). Given that fMRI measurements cannot yet be carried out in the real world, this is the closest we can get to representing the usual conditions of social affect and cognition, but perfect ecological validity can hardly be achieved by any fMRI experiment, including the paradigm used in this study. Consequently, there is a residual risk of decreased ecological validity in regard to the conclusions drawn from neuroscience experiments (see Mitchell, 2012, for a general discussion). Ecological validity should go along with an association of the measurement with everyday occurrences of the process of interest. Hence, research connecting neural activation with the psychological processes of interest assessed in daily life can be consulted in order to demonstrate that what we measure in the laboratory has a relevant relation to everyday life (Berkman et al., 2011). This approach is in line with a recent perspective paper on social cognition research that explicitly calls for examining what ToM tasks actually measure, for example by relating them to data stemming from actual interactions (Quesque and Rossetti, 2020). While EMA measures are not perfectly accurate measures of the actual interactions as suggested by Quesque and Rosetti, as they are not recorded during but right after an interaction, they are a step in that direction.

### 1.3 Ecological Momentary Assessment

Where ecological validation of fMRI research is intended, the criterion itself should be as ecologically valid as possible. Some researchers have applied retrospective measures to assess everyday behavior and its relation to neural activations (Dodell-Feder et al., 2014; Falk et al., 2010; Mahmood et al., 2013; Nikolova and Hariri, 2012). These retrospective measurements complement the neuroscience perspective with data from the individual’s life, but are still prone to cognitive biases associated with global self-reports.

Ecological Momentary Assessment (EMA), also referred to as ambulatory assessment or experience sampling, describes the measurement of psychological variables in daily life. For example, multiple self-reports per subject and day can be assessed using mobile technology. This methodology enables researchers to assess intrapsychic phenomena immediately and repeatedly in naturalistic settings and thereby capture subtle variation, as well as potential antecedents or consequences of the respective states. Hence, EMA is designed to assess the constructs of interest in everyday settings, in real-time, and over-time and thus to maximize ecological validity (Shiffman et al., 2008). These advantages help to avoid memory biases distorting the variables of interest and provide additional information about momentary variation and antecedents/consequences. Furthermore, EMA samples directly from everyday settings where psychological experience should be more salient and vivid than when recalled from memory (trait questionnaires), thus providing more nuanced information. This adds to the ecological validity of EMA and thus makes it a powerful methodology for the investigation of ecological validity of fMRI research.

To date, studies combing fMRI and EMA methodology are sparse, but pioneering work has been conducted in different fields, such as neural correlates of self-control and smoking cessation or reward system activity and positive affect (Berkman et al., 2011; Forbes et al., 2009; furthermore e.g.: Kluge et al., 2018; Lopez et al., 2016; Provenzano et al., 2018; Seidel et al., 2018; Wilson et al., 2014). Three studies examined social affect and cognition in an attempt to predict everyday prosocial behavior (Morelli et al., 2014; Rameson et al., 2012; Vekaria et al., 2020). The first study showed that mPFC activation was modulated by in-task experience of compassion and associated with mean daily helping (under cognitive load; Rameson et al., 2012). The second study showed that mean daily helping was associated with activity in a region identified as the septum (Morelli et al., 2014). A recent study further advanced these findings and showed that daily helping was associated with similar neural activation as in the previously described study, but the authors identified this region as the left bed nucleus of the stria terminalis (Vekaria et al., 2020). Taken together, these results represent an important first step towards validating neuroscience-based insight into empathy and related constructs by everyday data.

Nonetheless, some aspects remain to be investigated. First, in the small field of combined fMRI and EMA protocols in social affect and cognition research, related neural activation has been used to predict a conceptually proximal criterion variable (prosocial behavior; Ashar et al., 2017; Tusche et al., 2016) but not the constructs of interest themselves. It would thus be beneficial to evaluate whether neural activation predicts everyday occurrences of the same processes elicited by neuroscience paradigms. Second, so far, only combined activation clusters which do not discern socio-affective from socio-cognitive processes have been used to predict everyday behavior. This leaves distinct predictive power of activation patterns based on social affect and social cognition open to further investigation.

### 1.4 A Multimethod, longitudinal approach combining fMRI and EMA

In order to address these issues, we applied a multimethod approach combining four different sources of data: (1) behavioral responses from the EmpaToM task, which jointly assesses social affect and cognition, (2) corresponding neural activation using fMRI, (3) a social affect and cognition trait questionnaire, (4) and a subsequent 14-day EMA protocol assessing momentary social affect and cognition in social interactions. By using these different sources of data, we were able to investigate associations of laboratory and self-report trait measures with everyday data. This is especially important as these measures represent different access points to social affect and cognition. Behavioral measures of ToM, measuring the capacity to correctly infer others’ mental states, typically show little association with self-report trait measures of ToM (Murphy and Lilienfeld, 2019). This might reflect a distinction of ToM capacity, as assessed by behavioral paradigms, and ToM propensity, the proneness to engage in mentalizing, as assessed by trait questionnaires. Furthermore, the self-concept of how much one engages in perspective taking may differ from the actual propensity. Regarding empathy, associations of behavioral paradigms, measuring empathic responses in a situation, and questionnaires are stronger than those for ToM (around *r* = .5; Neumann et al., 2015). Behavioral measures of ToM and empathy are qualitatively different, as ToM measures are performance measures that assess whether one correctly infers others’ mental states, while affective responses are necessarily subjective. Behavioral measures of empathy are not performance measures and thus also rely on (momentary) self-report, which may increase associations with self-report trait questionnaires. Yet, behavioral and questionnaire measures are not perfectly correlated, which underlines a distinction of these approaches to the measurement of empathy.

Neural correlates of social affect and cognition, as assessed with the EmpaToM paradigm, have been shown to be associated with behavioral measures (Kanske, Böckler, et al., 2015). However, associations of these neural correlates with trait questionnaire measures as well as EMA-assessed everyday measures have not yet been probed. In this study, we examined associations between social affect and cognition trait measures, behavioral measures and neural correlates as well as their respective associations with everyday social affect and cognition.

Building on this data, we aimed to probe the ecological validity of fMRI-assessed neural correlates of social affect and cognition. To this end, we associated fMRI-assessed social affect and cognition-related neural activation with trait and everyday social affect and cognition assessed using EMA as an ecologically valid assessment of these constructs (Shiffman et al., 2008). Within meta-analytically defined regions of interest (ROIs), we expected ToM-related neural activation to predict behavioral, self-reported trait and everyday measures of social cognition and empathy-/compassion-related neural activation to predict behavioral, self-reported trait and everyday measures of social affect.

In addition to tests within the a-priori defined ROIs, we conducted exploratory whole-brain analyses to examine activation differences related to everyday social affect and cognition. Lastly, we were interested in contingencies of interaction characteristics (e.g., experienced affect during a social interaction) with experienced social affect and cognition and, more specifically, whether these contingencies are a function of individual neural activation related to social affect and cognition. For example, does being in a bad mood make it less likely to take another person’s perspective? And if so, is this within-person contingency stronger for individuals with less activation of the ToM network during social cognition? This approach takes into account that the propensity to engage in social affect and cognition (trait) may differ across situations (state) depending on the circumstances and that this contingency may differ across individuals (see e.g., Miskewicz et al., 2015). Building on this approach, we aimed to examine on an exploratory basis whether brain activation explained differences in these within-person contingencies of interaction characteristics and social affect and cognition.

## 2 Method

### 2.1 Participants

The final sample consisted of 122 participants (age mean = 25.5 years, *SD* = 6.9, age range: 18–57, 62 male). Exclusion criteria were age below 18 and above 60 years and non-suitability for MRI scanning. Data was acquired as part of a larger study on personality.^1^ Out of our initial sample, three participants were excluded from the analyses due to severe mental disorders for which complete remission cannot be assumed (in this sample: schizophrenia) or current intake of psychotropic medication. Due to technical issues or participant characteristics (e.g., anxiety in the scanner), 14 did not complete the scanning session and 13 were excluded from the analyses due to unusable data (anatomical abnormalities, artifacts and extensive movement). Another 16 participants did not provide sufficient EMA datasets (< 10 recordings in 14 days, *M* = 23.6, *SD* = 12.6), yielding the final sample of 122 participants. Although no directly comparable investigations have yet been conducted, earlier work suggest medium effect sizes (between *r* = .24 and *r* = .45) for the link of neural and everyday data (Rameson, 2011). A power analysis based on a power of .8 and a two-tailed *α* of .05 indicated that a sample of 85 participants would be needed to detect an effect of *r* = .3. Hence, when applying this estimate of effect size, our sample size should be sufficient. The project was approved by the Ethics Committee of Dresden University (Reference no 133042018) and all participants provided written informed consent. Participants were compensated with 120 Euros after completion of the study (laboratory session and 14-day EMA assessment).

### 2.2 Procedure and materials

**Fig. 1:**
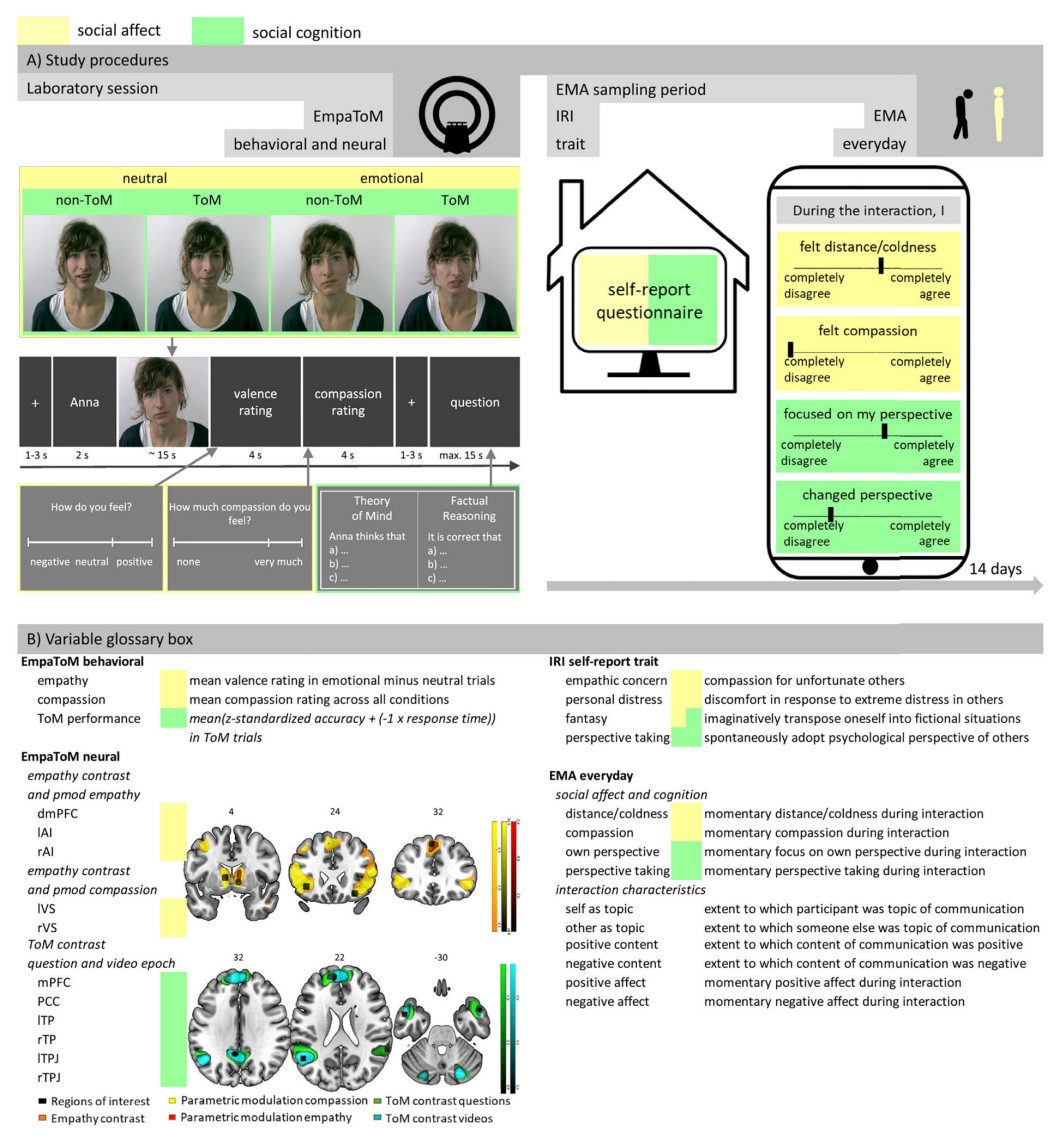
Illustration of the Study Procedures and Variable Glossary. *Note*. A) Laboratory session: Schematic illustration of an EmpaToM trial. Emotional and neutral videos that do or do not give rise to Theory of Mind questions (ToM requirement) are followed by empathy and compassion ratings and Theory of Mind and factual reasoning questions (adapted from Kanske, Böckler, et al., 2015). EMA sampling period: Exemplary screen with social affect and cognition items. B) Variable glossary box: Regions of interest displayed alongside the results of the second level contrasts the respective ROI activation parameters were derived from. IRI = Interpersonal Reactivity Index, pmod = parametric modulation, r = right, l = left, VS = ventral striatum, AI = anterior insula, dmPFC = dorsomedial prefrontal cortex, TPJ = temporoparietal junction, TP = temporal pole, PCC = posterior cingulate cortex.

Participants were selected based on an online screening in which suitability for MRI scanning and demographics were assessed along with other questionnaires not relevant to the hypotheses tested here. They were then invited to a lab and scanning session. Participants received instructions and performed practice trials of the EmpaToM task which they subsequently completed in the scanner. Furthermore, a structural and a resting-state scan and another behavioral task (outside of the scanner) were conducted. After the initial lab session, participants were instructed to the 14-day EMA assessment and given an assessment device (see below). The 14-day EMA period started on the following day for most participants. Participants also received a link to an online survey (see below) which they were asked to fill in at home (see Fig. 1 A for an overview).

#### 2.2.1 EmpaToM

This paradigm is designed to assess neural activation and behavioral performance related to social affect and cognition using a within-subjects design (Kanske, Böckler, et al., 2015). An item-analysis demonstrates good reliability of the task and both the neural as well as the behavioral measures of the EmpaToM have been validated with established tasks (Kanske, Böckler, et al., 2015; Tholen et al., 2020). We used one of the parallel versions consisting of optimized item sets identified by Tholen and colleagues (2020). Participants were instructed to empathize with individuals giving autobiographic narratives in short videos. The videos differed in emotionality of the content (negative or neutral) and in what question they gave rise to (ToM requirement; ToM vs non-ToM). That is, the narrators openly mentioned their thoughts and beliefs (no ToM requirement) or only alluded to what they were thinking and the participants had to deduce thoughts and beliefs (ToM requirement). This allowed asking mental state questions including first and second order perspective taking, true and false beliefs, preferences and desires, irony, sarcasm, metaphors, (white) lies, deception and faux pas (see also Tholen et al., 2020). Each of 48 trials consisted of a fixation cross (1–3 s) followed by the name of the individual in the video (1 s). After each video (~ 15 s), participants provided dimensional ratings of their current emotional state (from *negative* to *positive*; 4 s; valence rating) and how much compassion they felt for the person (from *none* to *very much;* 4 s; compassion rating). Lastly, after a fixation cross (1–3 s), a multiple choice question that required either mentalizing (e.g., “Susan thinks that…”, ToM condition) or factual reasoning (e.g., “It is true that…”; control condition) with three response options was presented. Participants had a maximum of 14 s to provide an answer (highlighted on the screen for another second). The task takes approximately 30 min to complete. Each participant completed one run. Figure 1 A displays an overview of one exemplary EmpaToM trial. The manipulation of emotionality and ToM requirement results in a 2 × 2 factorial design with four conditions each presented 12 times: (1) neutral – non-ToM, (2) neutral – ToM, (3) emotional – non-ToM, (4) emotional – ToM. To control for possible confounding effects of factor characteristics and sequence, each narrator appeared once in each condition and trial sequence was randomized. Behavioral empathy was operationalized by the mean valence rating in emotional minus neutral trials with higher values indicating a stronger attunement of the participants’ affect to the affect of the narrator in the video. Behavioral compassion was operationalized by the mean compassion ratings across all conditions. Behavioral ToM was operationalized by ToM performance (mean of z-standardized accuracy and the inverse of z-standardized response time in ToM-trials; Kanske, Böckler, et al., 2015).

#### 2.2.2 Ecological momentary assessment

At the end of the scanning session, participants were trained on the EMA procedure. The EMA protocol was implemented on a Samsung smartphone (Android platform) using custom survey software (similar to Pieper et al., 2018) and included the assessment of time- (six per day) and event-contingent recordings. In this study, we focused on the event-contingent recordings. For these, participants were instructed to complete a recording after every social interaction (personal or by phone/video; not text-based) with a duration of at least 10 minutes. Within this recording, participants provided information about interaction characteristics as well as social affect and cognition in form of slider bar items. To ensure comparable response behavior in similar subjective situations across participants, especially on the social affect and cognition items, participants completed five example questionnaires while an experimenter was present. Social affect and cognition, our primary dependent variables, were assessed by two items each, with one positive and one negative wording: “During the interaction, I felt compassion” / ”During the interaction, I felt distance/coldness” (social affect items) and “During the interaction, I changed perspective” / “During the interaction, I was focused on my own perspective” (social cognition items). These short items were intended as cues for social affect and cognition during the interaction as follows: Participants were instructed to consider compassion and perspective taking directed towards others (i.e., feeling warmth and care for someone else and taking someone else’s perspective) and also towards themselves (i.e., feeling warmth and care for oneself in the sense of self-compassion and considering other ways to think about a situation in the sense of metacognition) for their ratings. Whether these processes were actually directed towards others or towards the self was then assessed using separate items assessing the content of the interaction/communication (self as topic, other as topic). We used this self- vs. other-relatedness as well as valence of the content of the interaction/communication (positive, negative) and positive and negative affect (10 items, I-PANAS-SF; Thompson, 2007) as moderating variables in the situational analyses (see below). Note that the social affect items assessed compassionate affect sharing rather than pure empathy or pure compassion, thus including both, empathic affect sharing and feelings of warmth and care^2^.

Additionally, every prompt assessed the number of communication partners, duration of interaction, type of contact, content of the interaction/communication (future, past), feeling in situation (admired, belittled, comfortable, uncomfortable), state narcissistic grandiosity/vulnerability and state self-esteem. To limit the number of tests, we did not include these items as they were not of interest to the main research questions presented here.

Participants provided an average of 27.00 (*SD* = 11.58) event-based recordings throughout the 14-day sampling period. The number of provided recordings was not significantly related to any of the EMA variables relevant to our analyses (all *p*s > .05), except for a small negative association with distance/coldness, *r*(120) = −.21, *p* = .022. This indicates either that participants with higher average perceived distance/coldness experienced less social interactions, or were more likely to selectively report interactions where they perceived distance/coldness.

#### 2.2.3 Online survey

Following the initial lab session, participants completed an extensive online survey including trait measures of different aspects of personality (not analyzed here) and the Interpersonal Reactivity Index (IRI; Davis, 1980). This widely used 16-item self-report questionnaire on dispositional empathy has four subscales: (1) Perspective-Taking, the propensity to spontaneously adopt the psychological perspective of others (social cognition, e.g., “I believe that there are two sides to every question and try to look at them both.”), (2) Fantasy, the propensity to imaginatively transpose oneself into fictional situations (both social affect and social cognition, e.g., “Becoming extremely involved in a good book or movie is somewhat rare for me”, reverse coded), (3) Empathic Concern, the propensity to experience compassion for unfortunate others (social affect, e.g., “I often have tender, concerned feelings for people less fortunate than me”), and (4) Personal Distress, the propensity to experience discomfort in response to extreme distress in others (social affect, similar to empathy for others’ negative emotions in the sense of affect sharing, e.g.,” In emergency situations, I feel apprehensive and ill-at-ease.”). We applied the German version for which good reliability (Cronbachs *α* = .78) and external validity in form of associations with other questionnaires have been reported (Paulus, 2009). In this study, the sample means on most IRI subscales did not differ significantly from the norm (all *p*s >.05, one-sample *t*-test; Paulus, 2016). Only perspective taking (*t*(58) = −2.85, *p* = .006) in female participants as well as empathic concern (*t*(62) = −2.17, *p* = .034) and personal distress (*t*(62) = −2.41, *p* = .019) in male participants were significantly lower than the norm. However, these differences were all small (all below a third of the respective norm sample standard deviation). Thus, our sample can be considered widely representative of the general population regarding dispositional social affect and cognition.

#### 2.2.4 MRI Data Acquisition

Brain images were acquired on a 3 Tesla Siemens Tim Trio scanner (Siemens Healthcare GmbH, Erlangen) with a 32 channel head coil and built-in movement correction. A T1-weighted sequence (TR = 2300 ms; TE = 2.98 ms; TI = 900; flip angle = 9°; 176 sagittal slices; matrix size = 256 × 256; FOV = 256 mm; slice thickness = 1 mm) with a resulting voxel size of 1 × 1 × 1 mm was used for the structural images. A T2*-weighted echo planar imaging (EPI) sequence (TR = 2360 ms; TE = 27 ms, flip angle = 90°) was used for the functional images. Thirtyseven axial slices covering the whole brain (slice thickness = 3 mm, in-plane resolution = 3 × 3 mm, interslice gap = 1 mm, FOV = 210 mm; matrix size = 70 × 70) were acquired.

### 2.3 Data analysis

#### 2.3.1 fMRI data analysis

The imaging data were analyzed using SPM 12 (Wellcome Department of Cognitive Neurology, Institute of Neurology, London, United Kingdom) and are largely congruent to the analyses conducted by Kanske and colleagues (2015). Functional images were slice timing corrected and realigned to the mean image to correct for head motion. Each subject’s high resolution anatomical image was coregistered to the mean functional image and normalized. The resulting transformation matrix was then applied to normalize the functional scans to MNI space. Spatial smoothing with a Gaussian kernel of full-width half-maximum at 8 mm and a high-pass temporal filter with cutoff of 128 s to remove low frequency drifts were applied to the resulting functional images. To account for remaining motion artifacts after realignment, we used the robust weighted least squares (RWLS) toolbox (Diedrichsen and Shadmehr, 2005) which uses residual error estimates in first-level analysis to down-weight images with higher noise variance.

On the first level, a general linear model was fitted to model brain activation in response to each of the four conditions (neutral – nonToM, neutral – ToM, emotional – non-ToM, emotional – ToM) for video and question sequences respectively as well as the two rating periods. These regressors were convolved with a canonical hemodynamic response function (HRF). Movement parameters were added to the design matrix as nuisance regressors of no interest. Contrasting emotional to neutral videos (empathy contrast, on the second level) captures both empathy and compassion, as compassion may accompany empathy. Hence, brain activation in this contrast is not specific to empathy or compassion. To determine regions where activation tracked self-reported empathy or compassion, we built two first level models with only one regressor for all videos and one regressor for the parametric modulation of neural activation in the videos by (1) valence ratings (inversed so that higher values indicate more empathy) or (2) compassion ratings (cf. Kanske, Böckler, et al., 2015).

On the second level, two 2 × 2 (emotionality x ToM requirement) flexible factorial design models were fitted for random effects analysis. We modeled the video and the question epoch in separate models using the first level contrast images of the effects of each of the four conditions (neutral – non-ToM, neutral – ToM, emotional – non-ToM, emotional – ToM) during the respective epoch as regressors. Within these flexible factorial models, we modeled the empathy contrast (emotional vs. neutral videos) and the ToM contrasts (ToM vs. non-ToM videos and ToM vs. non-ToM questions) by applying linear weights to the parameter estimates. We performed two one group *t*-tests to test the parametric modulations of video-related activation by valence ratings (parametric modulation empathy) and compassion ratings (parametric modulation compassion). Corresponding to each of these five second level contrasts, two further second level models comprising EMA social affect (for the empathy contrasts) and cognition measures (for the ToM contrasts) as covariates were fitted. Thereby, we computed exploratory whole-brain estimates tracking areas where activation associated with the empathy and ToM contrasts and the parametric modulations interacted with EMA-assessed social affect and cognition measures. In other words, these models revealed areas where empathy-, compassion- or ToM-related neural activation differed as a function of mean everyday social affect and cognition. For each of these models, we tested the contrast that represents the effect of the interaction of the EMA covariate (social affect or cognition) with the respective factor (emotionality, ToM, or the parametric modulations) by applying linear weights to the parameter estimates. For visualization of results, contrasts were overlaid on a surface representation of the MNI canonical brain using MRI-croGL (https://www.mccauslandcenter.sc.edu/mricrogl/, 2019). As in previous work on the EmpaToM (Kanske, Böckler, et al., 2015), we applied a family wise error (FWE) corrected threshold of *p* < .05 and an extend threshold of k > 10 contiguous voxels to the analyses without covariates. Exploratory whole brain analyses including EMA covariates are reported at a more liberal threshold of *p* < .001 uncorrected with a cluster threshold of k > 10 contiguous voxels.

In order to avoid statistical overestimation of brain-behavior links (Kriegeskorte et al., 2010), we based our functional ROIs on peak activations (5 mm radius spheres) for the empathy and ToM contrasts from the largest EmpaToM dataset to date (Kanske, Böckler, et al., 2015). Furthermore, we extracted ROI activation parameters only for regions repeatedly shown to be activated in meta-analyses. These comprise (1) mPFC, PCC, bilateral TP and TPJ constituting a total of six social cognition ROIs (Bzdok et al., 2012; Schurz et al., 2014), (2) dmPFC and bilateral AI for empathy (Fan et al., 2011; Lamm et al., 2011) and (3) VS for compassion (Klimecki et al., 2013), constituting a total of five social affect ROIs. ROI activation parameters for the social cognition ROIs were extracted from contrast images for the ToM contrasts (ToM vs. non-ToM questions and ToM vs. non-ToM videos) and for social affect ROIs from the empathy contrast (emotional vs. neutral videos) and parametric modulations (empathy for empathy ROIs and compassion for compassion ROIs). While the ToM contrast derived from the question epoch represents deliberate ToM, the ToM contrast derived from the video epoch represents a more indirect, spontaneous measure of ToM that may more closely reflect ToM propensity. Therefore, for the ROI analyses, we used activation derived from both epochs. We used the REX toolbox (https://www.nitrc.org/projects/rex/, 2019) to extract ROI activation parameters.

All further analyses were conducted using R statistical software (R Development Core Team, 2013) and are considered significant at a threshold of *p* < .05 (uncorrected) unless explicitly stated otherwise. Paired-sample (empathy and ToM contrast) and one-sample (parametric modulations) *t*-tests were used to determine whether ROIs were significantly activated in the emotional or ToM versus neutral or non-ToM conditions, or as a function of trial-wise ratings (parametric modulations). Associations of ROI activation with EmpaToM behavioral outcomes, IRI trait measures and mean EMA social affect and cognition were tested by calculating bivariate Pearson’s correlations. As the main research question of this study, we hypothesized that social affect-related neural activation would predict everyday social affect and social cognition-related neural activation would predict everyday social cognition. Thus, for these hypotheses, we applied the Bonferroni-Holm correction, which adjusts the *p*-values according to the total number of ROIs used in each family of analyses (each of the four primary dependent variables), to correct for multiple comparisons (see e.g., Kanske, Schönfelder, et al., 2015).

#### 2.3.2 EMA data analysis

To address the nested nature of the EMA data, we fitted two-level multilevel linear models with situations (level 1) nested within participants (level 2). All models included random intercepts, so that mean levels of social affect and cognition were allowed to vary between participants, and random slopes, which means that the slope between an interaction characteristic and momentary social affect or cognition was allowed to vary between participants. For the exploratory analyses of within-person contingencies, in a first set of models, all EMA interaction characteristic variables were grand mean centered and entered individually as fixed and random effects. The models were fitted for the four dependent variables compassion, distance/coldness (social affect items), perspective taking and focus on the own perspective (social cognition items), respectively. With these models, we aimed to examine which interaction characteristics were associated with social affect and cognition. The proportion of between-person to total variance as expressed by the intraclass correlation was .36 for compassion, .25 for distance/coldness, .35 for perspective taking and .24 for focus on the own perspective. This means that substantial amounts of variance can be attributed to the between-person level.

To examine whether within-person contingencies were moderated by neural activation during social affect and cognition, we fitted a second set of models. For each of the within-person contingency models described above, we fitted ten (social affect) or twelve (social cognition) models by adding each of the ten (social affect) or twelve (social cognition) corresponding ROI activation variables individually as a level 2 predictor (fixed effect). Thus, we modeled each EMA social affect and social cognition variable as a function of each interaction characteristic and activation of each corresponding ROI. We focused on bivariate (one level 1 and one level 2 predictor) rather than multivariate models here as keeping activation in other regions constant is not a realistic assumption. All variables were *z*-standardized for this set of models. We did not conduct stepwise model selection, as we did not ask for a model that best describes the data, but were exclusively interested in the interaction term.

To test whether controlling for interaction characteristics accentuated the relevant associations of ROI activation parameters with EMA social affect and cognition, we built another random slope, random intercept multilevel model. This model comprised fixed effects for all six interaction characteristics, mPFC ROI activation (video epoch) and random effects for all six interaction characteristic variables nested within participants with EMA perspective taking as the dependent variable.

## 3 Results

To investigate whether neural responses predict everyday social affect and cognition, we tested associations with longitudinal EMA data. First, we briefly summarize the precursory results that replicate the behavioral and neural outcomes of the EmpaToM paradigm. Then, we report associations of the EMA social affect and cognition variables with behavioral and questionnaire data to explore the validity of our primary dependent variables. To address our primary research question, we describe associations of neural activation with everyday social affect and cognition. In a final step, we report the results of the situational contingency analyses that explore whether individual differences in neural activation moderate the association of interaction characteristics with everyday social affect and cognition.

### 3.1 Precursory analyses

#### 3.1.1 EmpaToM behavioral

The effects of emotionality of the videos and ToM requirement on rating measures and ToM performance largely replicate previous work on the EmpaToM (Kanske, Böckler, et al., 2015). As opposed to prior findings, performance was not better in ToM trials than in non-ToM trials. As ToM and non-ToM questions were carefully matched regarding difficulty, this might indicate that in the version of the task we used in this study (optimized selection of stimuli from originally five parallel versions that were all validated with other established tasks; Kanske, Böckler, et al., 2015; Tholen et al., 2020), difficulty matching between conditions worked well. Also, the mean age in the original EmpaToM study was higher than in the present study and aging seems to lead to stronger reductions in non-ToM than in ToM reasoning (Reiter et al., 2017). Overall, the results suggest that the behavioral measures were suitable indicators of the respective processes. Further details are reported in the supplementary material (Supplementary Tables S1 to S5 and supplementary Figure S4).

#### 3.1.2 EmpaToM neural

The effects of emotionality of the videos and ToM requirement as well as trial-wise ratings of valence (emotional state of the participant) and compassion (towards the actor in the video) on neural activation (empathy and compassion parametric modulations) closely resemble prior findings using the same paradigm (Kanske, Böckler, et al., 2015) and show activation in all regions implicated in social affect and cognition meta-analyses (Bzdok et al., 2012; Fan et al., 2011 Lamm et al., 2011; Schurz et al., 2014). Figure 1 B illustrates these results alongside the ROIs from which neural activation was extracted.

**Fig. 2:**
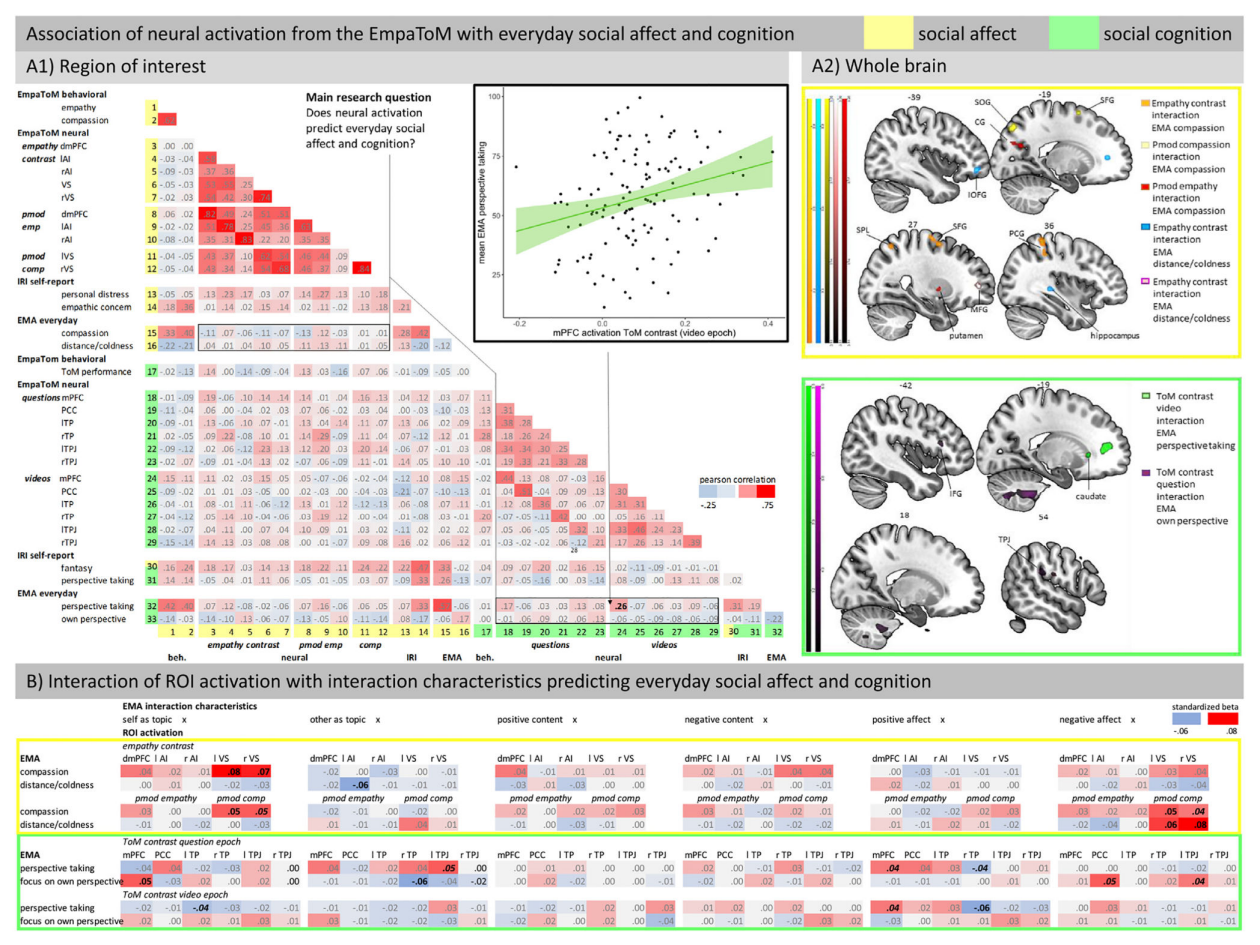
Illustration of the Results. *Note*. A1) Region of interest: Heatmap of bivariate Pearson’s correlations of all person-level variables. Correlations ≤ *r* = −.18 and ≥ *r* = .18 are significant (uncorrected *p*). Areas concerning our primary research question are highlighted with a black box. For the tests concerning our primary research question, we used the Bonferroni-Holm method to correct for multiple comparisons within each family of tests. Correlations significant after multiple testing correction are highlighted in bold. The significant correlation of mPFC activation (video epoch) and EMA mean perspective taking is depicted in the scatterplot. A2) Whole brain: Regions, where neural activation from the empathy contrast and parametric modulations was associated with everyday social affect and regions where neural activation from the ToM contrasts was associated with everyday social cognition. B) Interaction of ROI activation with interaction characteristics predicting everyday social affect and cognition: Heatmap of standardized beta-coefficients of the interaction term of multilevel models predicting momentary social affect and cognition by interaction characteristics and ROI activation. Significant coefficients are highlighted in bold, trends are highlighted in bold and italics. IOFG = inferior orbital frontal gyrus, SOG = superior occipital gyrus, CG = calcarine gyrus, SFG = superior frontal gyrus, SPL = superior parietal lobule, MFG = middle frontal gyrus, PCG = postcentral gyrus, IFG = inferior frontal gyrus, TPJ = temporoparietal junction, r = right, l = left, VS = ventral striatum, AI = anterior insula, dmPFC = dorsomedial prefrontal cortex, TP = temporal pole, PCC = posterior cingulate cortex, pmod = parametric modulation.

All social affect ROIs were significantly activated in the empathy contrast and in the parametric modulation analyses and all social cognition ROIs were significantly activated in the ToM contrasts (all *p*s < .001). In summary, activations of ROIs within the empathy network were correlated and activations of ROIs within the ToM networks (extracted from the video and question epoch, respectively) were correlated. At the same time, activations of ROIs within the empathy network were largely uncorrelated with activations of ROIs within the ToM network, supporting the distinction of these networks (see Figure 2 A1, Multitrait-Multimethod-Matrix and supplementary Figure S4).

#### 3.1.3 Validation of EMA measures with self-report trait and behavioral data

As the presentation of detailed results for associations between EMA measures and self-report as well as behavioral data is beyond the scope of this article, we will summarize these associations at the level of result patterns here. Detailed results are presented in Figure 2 (A1, Multitrait-Multimethod-Matrix) and supplementary Table S7.

EMA measures should be associated with the corresponding trait measures, as EMA assesses states corresponding to associated traits. Supporting this, both the social affect and the social cognition EMA measures correlated with the corresponding IRI measures. As expected, because both methods assess momentary self-reported affect, EMA social affect measures correlated with the corresponding behavioral measures. EMA and IRI social cognition measures did not correlate with the corresponding behavioral measure. This conforms to expectations, as previous work indicates that self-report and behavioral measures of social cognition diverge (see paragraph 1.4), potentially pointing to a distinction between perspective taking propensity (trait and state) and capacity (behavioral). Overall, these association patterns support the validity of the EMA measures and more strongly so for the EMA measures with positive wording (compassion, perspective taking) than for those with negative wording (distance/coldness, focus on own perspective).

#### 3.1.4 Validation of ROI activation with self-report trait and behavioral data

Social affect-related ROI activation was not significantly associated with corresponding behavioral measures (all *p*s > .05), but weakly associated with self-report trait measures. There was a small correlation of the IRI Personal Distress subscale with activation in the left AI, both extracted from the empathy contrast (*r* = .23, *p* = .011) as well as from the parametric modulation empathy contrast (*r* = .27, *p* = .003).

Social cognition-related ROI activation was weakly associated with behavioral measures. There was a small association of ToM performance with activation in the right TP, both extracted from the ToM contrast during questions (*r* = .28, *p* = .002) and videos (*r* = .20, *p* = .028). Social cognition-related ROI activation was not significantly associated with corresponding self-report trait measures (all *p*s > .05), except for a small association of the IRI Fantasy subscale with left TP activation from the question epoch (*r* = .21, *p* = .02). For details see Figure 2 (A1, Multitrait-Multimethod-Matrix).

### 3.2 Does neural activation predict everyday social affect and cognition?

#### 3.2.1 Association of ROI activation with EMA social affect and cognition

To examine whether neural activation was associated with everyday social affect and cognition, we extracted activation from the empathy contrast, parametric modulations and ToM contrasts in functionally defined (Kanske, Böckler, et al., 2015), meta-analytically confirmed ROIs from the social affect (dmPFC, AI and VS; Fan et al., 2011; Klimecki et al., 2013; Lamm et al., 2011) and social cognition (mPFC, PCC, TP and TPJ; Bzdok et al., 2012; Schurz et al., 2014) networks, respectively. These activations were used as predictors for everyday social affect and cognition and as moderators for within-person contingencies of interaction characteristics on social affect and cognition.

Activation in the mPFC ROI in the ToM contrast during the video epoch, possibly more closely reflecting spontaneous ToM in terms of ToM propensity, was significantly associated with mean EMA-assessed perspective taking (*r*= .26 *p* = .041, Bonferroni-Holm corrected, see Fig. 2, A1). EMA-assessed perspective taking was not associated with activation in any of the empathy ROIs, underlining the discriminant validity of this effect for social cognition-related brain activation. We did not compute a regression to demonstrate mPFC predictive validity above other laboratory or questionnaire measures, because mPFC activation was not associated with them. Yet, the association with EMA perspective taking indicates that mPFC activation explains unique variance in everyday perspective taking that was not explained by IRI trait measures.

This effect could be detected although EMA data originated from many, potentially very distinct situations and calculating person-level means of EMA variables aggregates over these. To further investigate the nature of this effect, we built a multilevel model predicting situation-level perspective taking by mPFC activation in the ToM contrast (video epoch), controlling for all six interaction characteristics. In this model, mPFC activation significantly predicted perspective taking to a greater extent, *b* = 44, *SE* = 12.75, *p* = <.001 (Satterthwaite approximation; Luke, 2017; see supplementary tables S8 and S9 for details on the models). To illustrate this, consider the following example of one covariate: The more positive affect a participant experienced during the interaction, the more did ToM-evoked neural activation in the mPFC predict everyday perspective taking (see supplementary Figure 2, displayed for illustrative purposes, interaction marginally significant). Note that the EMA compassion and perspective taking items were highly correlated in this study (see Fig. 2, A1). Spontaneous ToM-evoked neural activation in the mPFC may thus be more closely related to momentary perspective taking when individuals experience positive affect, potentially towards the communication partner.

None of the other ROIs were significantly associated with EMA mean social affect and cognition variables (all *p*s > .05, see Fig. 2, A1 for an overview).

#### 3.2.2 Whole-brain associations of neural activation with EMA social affect and cognition

To examine where in the brain neural activation was associated with everyday social affect and cognition, we conducted exploratory whole-brain analyses on interactions of EMA social affect and cognition variables with activation in the empathy contrast, ToM contrasts (question and video epochs), as well as parametric modulations. The results of these analyses indicate regions of the brain where individuals who experience more social affect and cognition in daily life show higher (or lower) neural activation in relation to empathy, compassion or ToM (see Fig. 2, A2 and Table 1).

Higher levels of everyday compassion were associated with (1) stronger empathy contrast activation in the right superior parietal gyrus (SPG), superior frontal gyrus (SFG) and postcentral gyrus (PCG), (2) stronger compassion-related activation (pmod compassion) in the left superior occipital (SOG) and superior frontal gyrus (SFG), and (3) stronger empathy-related activation (pmod empathy) in the left calcarine gyrus and the right putamen.

Higher levels of everyday distance/coldness were associated with (1) stronger empathy contrast activation in the left inferior orbital frontal gyrus (IOFG) and the right hippocampus and (2) stronger compassion-related activation in the right middle frontal gyrus (MFG).

Higher levels of everyday perspective taking were associated with stronger ToM contrast activation (video epoch) in the left caudate. Higher levels of everyday focus on the own perspective were associated with stronger ToM contrast activation (question epoch) in bilateral cerebellum, right TPJ and rolandic operculum as well as left inferior frontal gyrus (IFG).

#### 3.2.3 Predicting within-person situational contingencies by differences in neural activation

In an exploratory approach, we modeled associations between six EMA interaction characteristics and four social affect and cognition measures to test whether these within-person contingencies were moderated by between-person differences in ROI activation parameters. First, we examined which interaction characteristics were associated with momentary experience of social affect and cognition. All interaction characteristics were significantly associated with at least three of the four social affect and cognition measures. Furthermore, the strength and partly also direction of these within-person contingencies differed meaningfully between participants (see supplementary table S10 for results of the models and standard deviations of within-person contingencies). Next, we asked whether these differences in within-person contingencies were moderated by differences in social affect and cognition-related neural activation. Figure 2 (B) gives an overview of the strength of the interactions in these models. To give an example, participants reported more focus on the own perspective the more they themselves were topic of the communication and this effect was accentuated in participants with higher mPFC activation in the ToM contrast (question epoch, see supplementary Fig. S3). Put differently, mPFC activation predicted everyday focus on the own perspective more strongly when considering situations in which the participants themselves were topic of the communication.

When considering the overall pattern of results (see Fig. 2 B), these analyses suggest a moderating role of neural activation for within-person contingencies between the topic of the communication (oneself or someone else) as well as momentary affect (positive or negative) and experienced social affect and cognition in a social interaction. Put differently, social affect and cognition-related neural activation extracted from lab paradigms may not predict everyday social affect and cognition to the same extent in all situations.

## 4 Discussion

This study investigated the ecological validity of laboratory measures of social affect and cognition, specifically their fMRI-assessed neural correlates, by associating them with EMA-assessed everyday social affect and cognition.

Social affect and cognition are currently understood as two related, yet distinguishable routes to understanding others. Our results support this distinction, as associations between different data sources (behavioral and neural measures obtained in the lab, EMA data, and global self-reports) were observed within, but not between the two constructs. In accordance with this, the results of the analyses linking neural activation to everyday data differed between social affect and cognition.

First, we validated the EMA social affect and cognition variables used in this study by examining their association patterns with trait and behavioral measures. For social affect, behavioral, trait, and everyday measures showed small to moderate associations. It is important to note that behavioral measures of social affect also rely on self-report, as affect is necessarily subjective. In contrast, for social cognition, selfreport (trait and everyday) measures were interrelated, but unrelated to the behavioral measure; a pattern that resembles previous findings (Murphy and Lilienfeld, 2019; Neumann et al., 2015). This might reflect that the self-report measures assess propensity, or proneness to engage in social cognition, whereas the behavioral index measures social cognition capacity. The divergence between propensity and capacity has recently been discussed as a reason for low correlations between selfreport and behavioral measures, underlining the importance to consider this distinction also in fMRI research (Dang et al., 2020). Furthermore, one’s belief about own perspective taking capacity or propensity, as assessed in self-reports, may differ from actual propensity for perspective taking in everyday life. Thus, the lack of an association of the social cognition behavioral measure with everyday and trait social cognition in this study is not surprising. Consequently, for both the social affect and the social cognition variables, association patterns conformed to expectations, supporting the validity of the EMA measures used in this study.

### 4.1 Ecological validity of neural correlates of social affect and cognition

Results also differed for social affect and cognition regarding associations of neural correlates with everyday measures. For social cognition, activation in the mPFC, a region consistently associated with ToM (Bzdok et al., 2012), predicted everyday perspective taking, while empathy- and compassion-related neural activation did not predict everyday perspective taking. This finding further supports the distinction of social affect- and cognition-related neural networks identified in prior work (Bzdok et al., 2012; Fan et al., 2011; Kanske, Böckler, et al., 2015; Lamm et al., 2011; Schurz et al., 2014). Activation in the mPFC during the question epoch showed the same trend, but this effect did not survive multiple testing correction. ToM-related neural activation from the video epoch might more closely reflect ToM propensity as compared to ToM capacity in the question epoch, where mentalizing is directly demanded. EMA measures of social cognition assess propensity rather than capacity and may thus be more closely related to neural correlates of spontaneous mentalizing (ToM propensity). The relation of mPFC activation to everyday perspective taking was present even when aggregating over many, potentially very distinct situations. When controlling for interaction characteristics of the EMA recordings, this association was further accentuated. For example, mPFC activation had a stronger association with everyday perspective taking in social situations in which the participant experienced more positive affect. The observation of an association between neural activation and everyday events, which is significant even when aggregating over situations that probably deviate widely from the laboratory task environment, supports the ecological validity of the assessment of neural correlates of social cognition, and in particular those derived from the EmpaToM paradigm. As activation in the mPFC was not associated with behavioral and questionnaire data, this result represents unique variance explained by neural activation beyond behavioral and questionnaire indices. This result adds to emerging evidence supporting the predictive power of neuroimaging data for everyday experiences assessed in daily life (see e.g. Nikolova and Hariri, 2012). Although behavioral and self-report measures of social cognition were unrelated, neural activation in social cognition ROIs was also associated with the behavioral measure of social cognition. This suggests that behavioral and self-report indices captured different aspects of social cognition, which were both related to individual differences in neural activation, further supporting the ecological validity of neural correlates of cognition as assessed with lab-based paradigms.

At the same time, it needs to be noted that ROI activation predicted everyday perspective taking for only one of the six social cognition ROIs and only for the EMA item with the positive wording (“During the interaction, I changed perspective”). This shows that most of the ROI activation parameters we extracted were not predictive of everyday social cognition. As this study is the first to examine associations of neural correlates of social affect and cognition with everyday social affect and cognition, we chose a rather large set of ROIs (all of those reliably related to social cognition) and opted for an EMA assessment using both an item with a positive and an item with a negative wording. Higher intercorrelations of the EMA item with the positive wording with other social cognition measures suggest that this item may more closely reflect the construct of perspective taking than the item with the negative wording. This, as well as the results of this study regarding associations of ROI activation with everyday social cognition, may inform the design of EMA assessments and ROI selection in future studies. Nevertheless, a replication of the association of mPFC activation with everyday perspective taking is required to confirm this novel finding.

Within the social affect ROIs, empathy- and compassion-related neural activation did not predict everyday social affect and was also not related to behavioral and only weakly related to self-report trait measures of social affect. Yet, we do not consider this a contradiction to the ecological validity of neural correlates of social affect. In this study, we instructed participants to empathize with the narrators and they were asked to rate their compassion with the narrator after every trial. Therefore, we may have assessed deliberate rather than spontaneous social affect-related neural activation as opposed to spontaneous social affect assessed in the EMA protocol. Furthermore, the EmpaToM assesses empathy, potentially triggering and thus including other social emotions including compassion, but with a stronger focus on empathy, while our EMA protocol items on social affect assessed compassionate affect sharing with a stronger focus on compassion.

### 4.2 Implications of exploratory analyses

In addition to the ROI-guided analyses in this study, we conducted exploratory whole-brain analyses to identify regions where neural activation was associated with everyday social affect and cognition. These analyses yielded two interesting findings: The whole-brain analysis with EMA-assessed focus on the own perspective as a covariate underlines the relevance of the IFG for social cognition (see Fig. 2, A2), a region also previously described in ToM research (Bzdok et al., 2012; Schurz et al., 2014). The whole-brain analyses linking empathy- and compassion-related neural activation to everyday social affect and cognition show an interesting pattern of results: individuals who reported higher everyday distance/coldness showed more empathy- and compassion-related activation in prefrontal regions (MFG and IOFG), which are generally associated with executive functions or cognitive control (e.g., Yuan and Raz, 2014). Individuals who reported higher everyday compassion showed more empathy- and compassion-related activation in more posterior regions including the somatosensory cortex (PCG), a region associated with mirror neurons, which have been discussed as a mechanism underlying embodied affect sharing (e.g., Acharya and Shukla, 2012).

Lastly, we examined whether ROI activation moderated within-person contingencies between interaction characteristics and the experience of social affect and cognition in a social interaction. As this is a very new field of research, we had no a priori hypotheses regarding specific interaction characteristics, but conducted exploratory analyses encompassing different characteristics. As a consequence, individual results of these analyses should be interpreted with caution due to the resulting large number of tests. Yet, this study demonstrates the importance of certain interaction characteristics – above all the degree of self-relatedness of a social interaction – and can thus guide future studies building on these preliminary results. In daily life, psychological experience in social situations is subject to widely varying interaction characteristics and individual differences in neural activation might have varying impact on everyday experience as a function of context. Situations in the laboratory are more standardized/controlled than in everyday life. Thus, it is reasonable that neural activation extracted from lab paradigms may be more strongly associated with everyday social affect and cognition from situations that show characteristics similar to those in the lab. The situational contingency analyses in this study take a first step to identifying relevant interaction characteristics to be considered in future combined fMRI and EMA studies on social affect and cognition.

In the present study, as can be seen in the pattern of significant results grouped by interaction characteristics (see Fig. 2 B), this manifested especially in the extent to which the participants themselves or others were topic of the communication. This is an important observation, considering that most social neuroscience paradigms examine situations in which *others* are topic of the communication. For example, participants reported higher everyday focus on the own perspective the more they were topic of the communication and less focus on the own perspective the more others were topic of the communication. These effects were accentuated for participants with higher ToM-related mPFC and right TP activation (see Fig. 2 B and supplementary Figure S3). These findings might reflect higher adaptation of one’s own perspective to the perspective of the individual that is currently topic of the communication in individuals with higher ToM network activation.

Furthermore, the results implicate a role of momentary affect for the experience of social affect and cognition in a social situation. For example, associations between momentary positive affect and perspective taking depended on ToM network activation. Participants with higher ToM-related mPFC and PCC activation had stronger positive associations of positive affect and perspective taking, while participants with higher ToM-related right TP activation had weaker positive associations of positive affect and perspective taking (see Fig. 2 B and supplementary Figure S2). This underlines that components of the social affect and cognition networks, although all meta-analytically confirmed, should be examined individually, as individual functions of the components may differ. In a first exploratory approach, these analyses of the influence of between-person variables on within-person contingencies demonstrate the potential of combined fMRI and EMA protocols.

### 4.3 Limitations and future directions

Although the functional ROI approach we applied in this study is an important step towards preventing statistical overestimation of brain-behavior links (Kriegeskorte et al., 2010), it also has limitations. In the EmpaToM paradigm, participants were instructed to empathize with the individuals in the videos, thus it may not purely assess empathy propensity but to some extent deliberate empathy. Hence, our ROIs were based on results based on deliberate empathy (Kanske, Böckler, et al., 2015) while the peaks might be slightly different for spontaneous empathy. The exploratory whole-brain analyses we conducted to identify where in the brain social affect and cognition-related activation was associated with everyday social affect and cognition provide valuable information on potential ROIs for future research, for example the PCG for social affect or the IFG for social cognition (see Figure 2, A2).

Previous research has demonstrated that neural activation in empathic capacity conditions does not differ between high and low trait empathy individuals, while neural activation in empathic propensity conditions is higher in individuals with high trait empathy (Rameson, 2011). Thus, the instruction to empathize might have diminished individual differences in empathy and thus variance of empathy-related neural activation in our sample, thereby limiting the potential to demonstrate associations with everyday data. Furthermore, we assessed social affect by means of two items due to a lack of short state scales suitable for EMA. The associations of these items with trait and behavioral measures support their suitability for the assessment of social affect, more specifically compassion. Yet, the EmpaToM empathy contrast primarily captures neural correlates of empathy, which may be accompanied by compassion to varying degrees. Although neural correlates of these two aspects of social affect can be partly disentangled using parametric modulation, applying a specific EMA empathy measure, to match the assessment of neural correlates of empathy in the EmpaToM, might benefit finding associations of neural and everyday social affect measures. For example, some EMA studies have operationalized empathy by comparing affect ratings of two individuals (see e.g., De Ridder et al., 2015). The development and evaluation of specific short scales for empathy, compassion, and ToM for EMA would be beneficial to both, research on social affect and social cognition. Furthermore, we instructed participants to consider both compassion and perspective taking towards others as well as towards the self (self-compassion and meta-cognition) for the EMA ratings. Although this provides interesting information for further analyses, it may have led to an overshadowing of the relation to neural correlates as these solely rely on social affect and cognition towards others. This is also underlined by the results on within-person contingencies, which show that the extent to which someone else was topic of the communication was relevant for associations of neural activation to social affect and cognition. Also, EMA measures were distinct from the behavioral laboratory measures of social cognition because the EMA measures relied on self-report. Hence, we cannot know how much participants really successfully took others’ perspectives (which, again, would require a second-person assessment), while we do have objective behavioral measures from the laboratory task. Hence, future research might benefit from a separate assessment of self- and other-directed affect and cognition and second-person approaches (i.e., the interaction partner also provides EMA ratings) in EMA protocols.

As little is known about the associations of neural and everyday measures of social cognition, we analyzed associations between several regions of interest and EMA measures. This leads to a nuanced picture of brain-behavior relations but also to a large number of tests, which limits the conclusions that can be drawn from individual results, especially for the situational contingency analyses. While this study takes a first step in examining associations of activity in specific brain regions with everyday social affect and cognition, future studies might use multivariate approaches (e.g. multivoxel pattern analysis) to explore whether complex and non-linear voxelwise associations account for differences in social affect and cognition.Furthermore, we completed only one run of the EmpaToM paradigm, while it would have further improved the reliability of the task to complete multiple runs for each participant. Also, it needs to be noted that replicability of interindividual differences in neural activation is a necessary prerequisite for the interpretation and relevance of associations of neural activation with everyday behavior (Dang et al., 2020). Future studies should thus examine whether neural activation patterns related to social affect and cognition are stable across repeated measurements.

## 5 Conclusions

To our knowledge, this is the first study to demonstrate differential predictive validity of fMRI-assessed neural correlates of social affect and cognition for corresponding EMA-assessed everyday measures. The results support the ecological validity of fMRI-assessed neural correlates of social cognition, as assessed by the EmpaToM (Kanske, Böckler, et al., 2015). Furthermore, they underpin the relevance of distinguishing between social affect and cognition capacity and propensity in social neuroscience. Further research should focus on neural correlates of empathic propensity to predict everyday measures of empathy, as these most likely assess empathic propensity and not capacity. The results of our exploratory analyses demonstrate the importance of considering social interaction characteristics when studying associations between neural correlates and everyday measures of social affect and cognition and can thus inform future hypothesis-based research.

## Supplementary Material

Supplementary Materials Supplementary material associated with this article can be found, in the online version, at doi: 10.1016/j.neuroimage.2020.117624.

## Figures and Tables

**Table 1:** Activation Peaks for Interactions of Empathy, Compassion and ToM During the EmpaToM With EMA Social Affect and Cognition Covariates

	H	CS	T	MNI Coordinates		
				x	y	z
Interaction of empathy contrast with EMA						
More activation with more everyday compassion						
Superior frontal gyrus	R	50	4.016	27	−9	66
Postcentral gyrus	R	16	3.695	36	−42	63
Superior parietal lobule	R	29	3.624	24	−63	57
Postcentral gyrus	R	25	3.603	30	−42	51
More activation with more everyday distance/coldness Inferior orbital frontal gyrus	L	17	3.956	−39	48	−9
White matter	L	18	3.934	−24	42	6
Hippocampus	R	11	3.800	36	−33	0
Less activation with more everyday distance/coldness Inferior occipital gyrus	L	21	−3.912	−36	−69	−3
Interaction of compassion parametric modulation with EMA						
More activation with more everyday compassion						
Superior occipital gyrus	L	41	4.316	−21	−78	45
Superior frontal gyrus	L	12	3.921	−18	3	66
More activation with more everyday distance/coldness Middle frontal gyrus	R	22	3.529	27	48	6
Interaction of empathy parametric modulation with EMA						
More activation with more everyday compassion Calcarine gyrus	L	22	3.852	−18	−69	24
Putamen	R	13	3.834	30	0	3
Interaction of ToM contrast (questions) with EMA						
More activation with more everyday focus on own perspective Cerebellum	L	317	4.591	−21	−42	−45
Cerebellum	L		4.434	−21	−72	−48
Cerebellum	R		4.071	3	−63	−42
Rolandic operculum	R	22	4.349	48	−15	21
Cerebellum	L	21	4.101	−36	−69	−24
TPJ - superior temporal gyrus	R	11	3.812	60	−57	15
Cerebellum	R	21	3.809	36	−51	−42
Cerebellum	R	43	3.688	18	−48	−39
TPJ - supramarginal gyrus	R	12	3.681	54	−30	24
Inferior frontal gyrus (pars triangularis)	L	12	3.560	−45	24	15
Less activation with more everyday perspective taking Angular gyrus	L	14	−4.508	−33	−57	30
PCC - Posterior cingulate cortex	R	13	−3.858	6	−48	30
Interaction of ToM contrast (videos) with EMA						
More activation with more everyday perspective taking White matter	L	80	4.593	−21	42	9
Caudate	L	10	3.601	−18	24	0
Less activation with more everyday perspective taking Cerebellum	L	15	−3.654	−30	−57	−27

*Note*. ToM = Theory of Mind, EMA = Ecological Momentary Assessment, MNI = Montreal Neurological Institute standard brain, H = hemisphere, Cs = cluster size in number of voxels, TP = temporal pole, TPJ = temporoparietal junction, AI = anterior insula, PCC = posterior cingulate cortex.

## Data Availability

The data and code that support the findings of this study are available in the Open Science Framework at https://osf.io/f7zcr/.

## Footnotes

1 SINA – Self and Interpersonal Study of Narcissism. Participants were selected for both, high and low, narcissistic grandiosity and vulnerability, based on an online screening. As narcissism is associated with empathy and ToM (Vonk, Zeigler-Hill, Mayhew, & Mercer, 2013) and our sample ranges widely across the narcissism dimension, this adds to variability of these variables in our sample.

2 Following pilot experiments, the assessment of compassionate affect sharing seemed more in line with layperson conceptions of empathy (German: Mitgefühl) and thus more feasible for self-ratings. In contrast, rating “pure” empathy (in terms of affect sharing with or without warmth and care) seemed hard to accomplish in everyday situations, as empathy and compassion are not commonly perceived as different processes by untrained raters.
