# Supplementary material for: Brain activation during social cognition predicts everyday perspective-taking: A combined fMRI and ecological momentary assessment study of the social brain
Source: Neuroimage. Author manuscript; Available in PMC 2021 Jul 19. (PMC7611322; doi:10.1016/j.neuroimage.2020.117624)
Supplement: Supplementary Materials — Supplementary material associated with this article can be found, in the online version, at doi: 10.1016/j.neuroimage.2020.117624. [file EMS130373-supplement-Supplementary_Materials.pdf]

Brain Activation During Social Cognition Predicts Everyday Perspective-Taking: A Combined fMRI and Ecological Momentary Assessment Study of the Social Brain. Supplementary Material

1. Supplementary Methods

1.1 Materials

1.1.1 EmpaToM

Instructions in original language and translated to English:

„In diesem Experiment wollen wir untersuchen, wie es Menschen gelingt, sich in Andere hineinzuversetzen und ihre Gedanken und Gefühle nachzuvollziehen.

Dazu werden wir Ihnen eine Auswahl von kurzen Videos zeigen. In diesen Videos berichten Personen von Ereignissen aus ihrem Leben. Diese Videos wurden so ausgewählt, dass sie besonders emotional bewegende Ereignisse, aber auch ganz Alltägliches beinhalten. Wir bitten Sie, sich dabei in die Person in dem Video hineinversetzen.

Nach jedem Video kommt eine Rating-Skala, auf der Sie einschätzen sollen, wie Sie sich nach diesem speziellen Video fühlen. Bitte geben Sie an, ob Sie sich eher positiv (z.B. glücklich, fröhlich, zufrieden) oder eher negativ (z.B. traurig, ängstlich, ärgerlich) fühlen. Dann kommt eine zweite Rating-Skala, auf der Sie einschätzen sollen, wie viel Mitgefühl Sie für die Person in dem Video fühlen. Mit Mitgefühl sind Gefühle wie Zuwendung, Fürsorge, Wärme und Wohlwollen gemeint.

Als nächstes wird Ihnen eine Frage zu dem Video gestellt. Es werden auch immer drei Antwortmöglichkeiten gezeigt, von denen Sie eine auswählen sollen. Manchmal kann es sein, dass Sie den Eindruck haben, dass mehrere Antworten richtig sind. Dann wählen Sie bitte die Antwort aus, die am besten passt. Im Experiment haben Sie dann 15 Sekunden Zeit um eine Antwort auszuwählen, das ist genügend Zeit um alle Antwortoptionen zu lesen.“

“In this experiment we want to investigate how humans manage to empathize with others and to comprehend their thoughts and emotions. To this aim, we will show you a choice of short videos. In these videos individuals will tell you about events from their lives. Those videos were chosen to contain particularly emotionally moving as well as ordinary events. We kindly ask you to empathize with the person in the video.

After every video there is a rating scale on which you are supposed to rate how you feel after this specific video. Please rate whether you feel rather positive (e.g. happy, joyful, content) or rather negative (e.g. sad, fearful, angry). Then, there is a second rating scale on which you are supposed to

rate how much compassion you feel towards the person in the video. Compasson includes feelings such as affection, care, warmth and benevolence.

Next, you will be asked a question about the video. There will always be three response options, out of which you are supposed to choose one. Sometimes you may feel that several response options are correct. In this case, please choose the response option that fits best. During the Experiment, you have 15 seconds to choose an answer, which is enough time to read all response options."

1.2 Data Analysis

*1.2.1 Behavioral data analysis.*Behavioral data were analyzed using R statistical software (R Development Core Team, 2013). To test whether ratings (valence and compassion rating) and performance measures (response time, accuracy and performance composite) in the EmpaToM depended on the two within-subject factors emotionality of video (neutral vs negative) and ToM requirement (nonToM vs ToM), we set up five repeated measures analyses of variance (ANOVA, one for each dependent variable).

2. Supplementary Results

2.1 Preliminary Analyses

*2.1.1 EmpaToM behavioral.*After emotional compared to neutral videos, participants reported more negative affect , $F(1, 121) = 619.9$, $p < .001$, $\eta2 = 0.709$, and more compassion , $F(1, 121) = 412.5$, $p < .001$, $\eta2 = 0.439$, as in previous work on the EmpaToM (Kanske, Böckler, Trautwein, & Singer, 2015). ToM requirement had an effect of negligible effect size on both rating measures (see supplementary tables S 1 and S 2 for detailed results). Effect sizes of both emotionality and ToM requirement on performance measures were very small (see supplementary tables S 3, S 4 and S 5 for detail on the performance composite, response time and accuracy). This conforms with expectations, as ToM and non-ToM questions were carefully matched regarding difficulty.

*2.2.1 EmpaToM neural.*Viewing negative versus neutral videos (empathy contrast) yielded activation in a network comprising the bilateral AI, TPJ, caudate nucleus including VS, dmPFC, PCC, middle frontal gyrus and cerebellum. Within regions implicated in social affect and cognition, activation in the left AI, TPJ and right VS and TP varied parametrically with the valence ratings.

Comparing ToM and non-ToM questions (ToM contrast) yielded activation in a network comprising the bilateral TP, TPJ extending into medial temporal gyrus, mPFC extending into ACC, superior precuneus/PCC and cerebellum (see supplementary table S6). Our results on the empathy and ToM contrasts closely resemble prior findings using the same paradigm (Kanske et al., 2015).

2.2 Does neural activation predict everyday social affect and cognition?

2.2.1 Predicting within-person situational contingencies by differences in neural activation. Regarding the prediction of momentary experience of compassion, there was an interaction between the extent to which the participant was the topic of the communication and left VS (b = 0.41, SE = 0.12, p = .001), as well as right VS (b = 0.45, SE = 0.17, p = .012) activation in the empathy contrast. Participants with higher VS activation in the empathy contrast reported less compassion the less they were topic of the communication while participants with lower VS activation did not show this association (see supplementary Figure 1).

Regarding the prediction of momentary experience of distance/coldness, there was an interaction between the extent to which someone else than the participant was topic of the communication and left AI activation in the empathy contrast (b = -0.26 , SE = 0.12, p = .032). Participants with higher left AI activation in the empathy contrast reported lower levels of distance/coldness the more others were topic of the communication, while participants with lower left AI activation reported similar levels regardless of how much others were topic of the communication (see supplementary Figure S 2).

Regarding the prediction of momentary experience of perspective-taking, positive affect interacted with ToM network activation (mPFC, PCC and right TP, all ps < .1), tending towards significance. Participants with higher activation in mPFC and PCC reported considerably more perspective-taking when their affect was more positive, while this effect was negligible in participants with less ToM related activation in these regions. The interaction showed the reverse pattern for right TP activation (see supplementary Figure S 3).

Regarding the prediction of momentary focus on the own perspective, there was an interaction between mPFC activation in the ToM contrast and the extent to which the participant was topic of the communication (b = 0.25, SE = 0.11, p = .027). Participants reported more focus on the own perspective the more they were topic of the communication and this effect was accentuated in participants with higher mPFC activation in the ToM contrast (see supplementary Figure S 4). A similar pattern is reflected also in the interaction of right TP activation with the extent to which someone else but the participant was topic of the communication (b = 0.22, SE = 0.1, p = .035). Participants reported less focus on the own perspective the more others were topic of the communication and this effect was accentuated in participants with higher right TP activation in the ToM contrast (see figure 4 in supplements).

Supplementary Tables and Figures

Supplementary Table S 1

Repeated measures ANOVA summary table for affect (EmpaToM valence rating)

Predictor	df_{Num}	df_{Den}	SS_{Num}	SS_{Den}	F	p	η^2_g
(Intercept)	1	121	135.17	89.55	182.65	.000	.40
Emo	1	121	495.32	96.68	619.92	.000	.71
ToM	1	121	1.50	7.47	24.37	.000	.01
Emo x ToM	1	121	0.29	7.67	4.52	.035	.00

Note. df_{Num} indicates degrees of freedom numerator. df_{Den} indicates degrees of freedom denominator. SS_{Num} indicates sum of squares numerator. SS_{Den} indicates sum of squares denominator. η^2_g indicates generalized eta-squared. Emo = Emotionality, ToM = Theory of Mind Requirement.

Supplementary Table S 2

Repeated measures ANOVA summary table for compassion (EmpaToM compassion rating)

Predictor	df_{Num}	df_{Den}	SS_{Num}	SS_{Den}	F	p	η^2_g
(Intercept)	1	121	5534.78	408.20	1640.63	.000	.91
Emo	1	121	446.20	130.89	412.49	.000	.44
ToM	1	121	3.20	14.29	27.10	.000	.01
Emo x ToM	1	121	0.00	13.30	0.02	.878	.00

Note. df_{Num} indicates degrees of freedom numerator. df_{Den} indicates degrees of freedom denominator. SS_{Num} indicates sum of squares numerator. SS_{Den} indicates sum of squares denominator. η^2_g indicates generalized eta-squared. Emo = Emotionality, ToM = Theory of Mind Requirement.

Supplementary Table S 3

Repeated measures ANOVA summary table for EmpaToM response time during questions

Predictor	df_{Num}	df_{Den}	SS_{Num}	SS_{Den}	F	p	η^2_g
(Intercept)	1	120	34153216537	1038582951	3946.13	.000	.97
Emo	1	120	3754711	47105423	9.57	.002	.00
ToM	1	120	556637	50777970	1.32	.254	.00
Emo x ToM	1	120	17819129	57103073	37.45	.000	.01

Note. df_{Num} indicates degrees of freedom numerator. df_{Den} indicates degrees of freedom denominator. SS_{Num} indicates sum of squares numerator. SS_{Den} indicates sum of squares denominator. η^2_g indicates generalized eta-squared. One participant excluded from analysis due to missing values on response time in one condition. Emo = Emotionality, ToM = Theory of Mind Requirement.

Supplementary Table S 4

Repeated measures ANOVA summary table for EmpaToM accuracy in questions

Predictor	df_{Num}	df_{Den}	SS_{Num}	SS_{Den}	F	p	η^2_g
(Intercept)	1	121	206.57	8.18	3056.99	.000	.93
Emo	1	121	0.17	2.71	7.40	.007	.01
ToM	1	121	0.44	2.92	18.24	.000	.03
Emo x ToM	1	121	0.37	2.18	20.74	.000	.02

Note. df_{Num} indicates degrees of freedom numerator. df_{Den} indicates degrees of freedom denominator. SS_{Num} indicates sum of squares numerator. SS_{Den} indicates sum of squares denominator. η^2_g indicates generalized eta-squared. Emo = Emotionality, ToM = Theory of Mind Requirement.

Supplementary Table S 5

Repeated measures ANOVA summary table for EmpaToM performance

Predictor	df_{Num}	df_{Den}	SS_{Num}	SS_{Den}	F	p	η^2_g
(Intercept)	1	120	0.02	209.14	0.01	.915	.00
Emo	1	120	0.00	26.00	0.00	.954	.00
ToM	1	120	0.01	22.06	0.03	.859	.00
Emo x ToM	1	120	0.00	21.48	0.01	.922	.00

Note. df_{Num} indicates degrees of freedom numerator. df_{Den} indicates degrees of freedom denominator. SS_{Num} indicates sum of squares numerator. SS_{Den} indicates sum of squares denominator. η^2_g indicates generalized eta-squared. One participant excluded from analysis due to missing values on response time in one condition. Emo = Emotionality, ToM = Theory of Mind Requirement.

Supplementary Table S 6

Activation peaks for empathy, compassion and ToM during the EmpaToM

				MNI Coordinates		
	H	CS	T	x	y	z
Emotionally negative > neutral videos						
Dorsomedial prefrontal cortex	L	885	11,849	3	48	33
Dorsomedial prefrontal cortex	L		9,838	-3	33	48
Superior frontal gyrus	L		7,162	-21	54	27
TPJ - supramarginal gyrus	L	372	11,394	-57	-51	36
TPJ - middle temporal gyrus	L		6,004	-39	-66	21
TPJ - supramarginal gyrus	R	658	10,047	57	-48	33
Middle occipital gyrus	R		6,341	36	-84	9
AI - inferior frontal triangularis	L	810	9,839	-48	21	6
Middle frontal gyrus	L		7,837	-42	9	48
AI - inferior frontal orbitalis	L		7,438	-30	24	-3
Striatum - caudate nucleus	L	519	9,663	-9	3	12
Striatum - caudate nucleus	R		9,197	12	6	12
White matter	L		5,317	-3	-6	-9
Cerebellum	L	190	9,549	-18	-78	-33
Cerebellum	R	230	9,414	18	-78	-30
AI - inferior frontal orbitalis	R	540	8,806	48	24	-3
Middle frontal gyrus	R		7,092	48	18	45
AI - inferior frontal orbitalis	R		6,221	30	21	-12
Posterior cingulate cortex	R	424	8,743	6	-57	36
Lingual gyrus	L	70	7,810	-9	-78	-3
Inferior temporal gyrus	R	26	6,807	54	-24	-12
TP - inferior temporal gyrus	R	14	6,204	51	3	-30
Middle cingulate cortex	L	11	5,479	0	-21	42
Emotionally neutral > negative videos						
Superior temporal gyrus	L	1172	-24,296	-57	-15	6
Superior temporal gyrus	R	893	-23,921	57	-12	3
Gyrus rectus	L	125	-7,124	-6	24	-18
Inferior temporal gyrus	L	10	-5,990	-57	-48	-15
Postcentral gyrus	L	28	-5,858	-18	-45	75

Postcentral gyrus	R	54	-5,745	45	-36	51
Postcentral gyrus	L	14	-5,728	-54	-6	45
Calcarine gyrus	L	10	-5,676	-6	-99	9
Middle cingulate cortex	R		-5,263	3	-36	33
Parametric modulation empathy						
More activation with more reported empathy						
dmPFC - superior medial gyrus	L	393	8.974	3	45	33
Superior temporal gyrus	R	339	8.415	57	-45	27
Supramarginal gyrus	L	303	8.368	-60	-51	36
Middle temporal gyrus	L	303	6.357	-45	-69	18
Precuneus	R	244	8.092	3	-54	42
Cerebellum	R	117	7.166	18	-81	-33
Cerebellum	L	73	6.948	-18	-81	-33
Caudate Nucleus	R	38	6.134	9	6	12
Inferior frontal triangularis	L	26	5.759	-51	18	6
Middle frontal gyrus	L	17	5.377	-39	9	48
Inferior frontal triangularis	R	13	5.175	51	24	6
Less activation with more reported empathy						
Superior temporal gyrus	R	569	-15.885	60	-9	3
Superior temporal gyrus	L	886	-15.059	-54	-18	6
Superior temporal gyrus	L	886	-8.615	-51	-36	18
Parametric modulation compassion						
More activation with more reported compassion						
dmPFC - Superior medial gyrus	L	653	9.720	-3	48	33
Superior medial gyrus	L	653	7.531	-6	30	48
Supramarginal gyrus	L	282	8.717	-54	-54	30
AI - Inferior orbital frontal gyrus	L	714	8.586	-42	30	0
Middle frontal gyrus	L	714	7.079	-42	15	45
Cerebellum	R	265	8.236	27	-72	-33
AI - Inferior orbital frontal gyrus	R	220	7.987	45	30	-3
Inferior orbital triangularis	R	220	6.296	57	24	15
Precuneus	L	256	7.871	0	-54	39
Superior temporal gyrus	R	225	7.798	63	-51	24
Cerebellum	L	172	7.774	-18	-78	-36
VS - Caudate nucleus	R	577	7.689	12	6	12
VS - Caudate Nucleus	L	577	6.987	-9	6	15
Location not in atlas		577	6.812	0	-24	3
Lingual Gyrus	L	60	6.214	-9	-78	-3
Superior frontal gyrus	L	31	5.900	-18	60	24
Cerebellum		10	5.204	0	-45	-18
Less activation with more reported compassion						
Superior temporal gyrus	R	396	-12.191	57	-12	3
Superior temporal gyrus	L	612	-11.469	-57	-18	6
ToM > nonToM questions						
Posterior cingulate cortex	L	1350	18.878	-6	-54	36
Cerebellum	R	212	18.750	27	-78	-33
Cerebellum	R		5.154	21	-63	-21
Cerebellum	L	101	16.405	-27	-81	-33
TPJ - superior temporal gyrus	L	1574	16.001	-48	-57	24
TP - medial temporal pole	L		14.990	-51	6	-30

Inferior orbital frontal gyrus	L		10.569	-48	30	-6
Medial prefrontal cortex	L	842	15.699	-6	54	33
Medial prefrontal cortex	L		8.899	-9	42	51
Dorsomedial frontal gyrus/supplementary motor area	L		8.271	-6	15	63
TP - medial temporal pole	R	1095	14.858	51	9	-33
Middle temporal gyrus	R		13.215	51	-33	0
TPJ - superior temporal gyrus	R		7.091	63	-45	18
Inferior orbital frontal gyrus	R	31	7.152	54	30	-3
Middle cingulate cortex		37	6.991	0	-15	39
Middle orbital gyrus	L	25	6.421	-3	60	-9
Cerebellum	R	46	6.212	3	-57	-39
Caudate nucleus	L	13	6.138	-9	9	18
Cerebellum		57	5.905	0	-63	-18
Lingual gyrus	L	21	5.687	-6	-75	-3
Precentral gyrus	L	14	5.387	-51	-6	45
Cuneus	R	16	5.240	15	-96	18
nonToM > ToM questions						
Angular gyrus	R	992	-25,540	39	-66	45
Inferior parietal lobule	R	992	-21,077	51	-45	51
Precuneus	R	992	-6,495	9	-69	48
Middle occipital gyrus	L	1087	-25,491	-33	-69	42
Inferior parietal lobule	L	1087	-15,178	-45	-48	48
Middle cingulate gyrus	R	1350	-22,943	0	-33	36
Calcarine gyrus	L	1350	-18,432	-9	-54	12
Precuneus	R	1350	-13,890	12	-54	15
Inferior temporal gyrus	L	1574	-22,935	-57	-51	-12
Superior frontal gyrus	R	2812	-22,287	27	18	54
Middle frontal gyrus	R	2812	-17,816	48	36	24
Middle frontal gyrus	L	2812	-17,301	-27	15	54
Inferior temporal gyrus	R	1094	-18,333	60	-45	-12
Inferior temporal gyrus	R	1094	-5,128	57	-21	-21
Inferior frontal triangularis	L	791	-13,572	-48	36	18
Inferior frontal opercularis	L	791	-10,887	-42	9	33
Inferior frontal orbitalis	L	791	-8,767	-30	33	-12
Fusiform gyrus	L	227	-12,571	-27	-39	-12
Cerebellum	L	117	-11,086	-36	-66	-42
Parahippocampal gyrus	R	64	-8,536	27	-30	-18
Cerebellum	L	56	-8,465	-9	-78	-27
Dorsal anterior cingulate	L	17	-5,831	-3	0	30
ToM > nonToM videos						
TPJ - middle temporal gyrus	L	288	10,950	-45	-54	24
Posterior cingulate cortex	L	236	10,907	-6	-51	39
Medial prefrontal cortex	L	488	9,557	-3	54	30
Superior medial gyrus	L	488	5,525	-6	45	48
Cerebellum	R	127	9,229	27	-84	-36
TP - middle temporal gyrus	L	320	9,113	-54	6	-30
Middle temporal gyrus	L	320	8,332	-57	-24	-6
Middle temporal gyrus	L	320	5,227	-54	-42	3
Middle occipital gyrus	R	626	9,031	27	-93	6
Inferior occipital gyrus	R	626	8,429	39	-81	-6
Cerebellum	R	626	5,663	36	-54	-18
TP - medial temporal pole	R	75	7,535	54	9	-27

Inferior occipital gyrus	L	644	7,504	-33	-84	-3
Cerebellum	L	644	6,912	-27	-81	-33
Calcarine gyrus	L	644	6,417	-9	-96	6
Inferior frontal orbitalis	L	25	6,452	-48	27	-9
Caudate nucleus	L	13	6,126	-9	6	15
Middle temporal gyrus	R	56	6,125	48	-21	-9
Middle temporal gyrus	R	19	5,849	54	-36	0
Inferior frontal triangularis	L	13	5,070	-51	21	9
nonToM > ToM videos						
Inferior temporal gyrus	L	147	-10,352	-54	-54	-9
Middle cingulate cortex	R	215	-9,710	0	-33	36
Middle occipital gyrus	L	397	-8,750	-36	-84	33
Middle occipital gyrus	L	397	-6,766	-30	-63	42
Inferior parietal lobule	L	397	-6,761	-48	-42	42
Inferior frontal triangularis	L	159	-7,733	-45	36	15
Inferior temporal gyrus	R	75	-7,223	60	-45	-12
Middle occipital gyrus	R	172	-7,170	42	-75	36
Supramarginal gyrus	R	172	-5,442	51	-45	48
Parahippocampal gyrus	L	50	-6,709	-30	-39	-9
Middle frontal gyrus	L	84	-6,472	-24	12	51
Calcarine gyrus	L	84	-6,419	-15	-57	18
Middle cingulate cortex	L	36	-6,218	-3	-6	27
Precuneus	R	53	-5,985	15	-54	21
Middle frontal gyrus	R	28	-5,848	27	12	57
Rectal gyrus	L	17	-5,601	-9	24	-15
Inferior frontal orbitalis	L	13	-5,413	-36	39	-12
Middle frontal gyrus	R	16	-5,094	48	48	6

Note. ToM = Theory of Mind, H = Hemisphere, CS = Cluster Size in number of voxels, MNI = Montreal Neurological Institute brain template, R = Right, L = Left, AI – Anterior Insula, VS – Ventral Striatum, TP = Temporal Pole, TPJ = Temporoparietal Junction

Supplementary table S 7

Means, standard deviations, and correlations with confidence intervals of all person-level variables

	Variable	*M*	*SD*	1	2	3	4	5	6	7	8	9	10	11	12	13	14	15	16	17	18	19	20	21	22	23	24	25	26	27	28	29	30	31	32
**EmpaToM behavioral**																																			
1	Empathic responding	2.01	0.89																																
2	Compassion	3.37	0.92	.62** [.49, .72]																															
**ROIs empathy contrast**																																			
3	dmPFC	0.14	0.19	.00 [-.17, .18]	-.00 [-.18, .17]																														
4	lAI	0.10	0.19	-.03 [-.21, .15]	-.04 [-.21, .14]	.58** [.44, .68]																													
5	rAI	0.06	0.16	-.09 [-.26, .09]	-.03 [-.20, .15]	.37** [.21, .51]	.36** [.20, .51]																												
6	lVS	0.13	0.19	-.05 [-.22, .13]	-.03 [-.21, .14]	.53** [.39, .65]	.55** [.41, .66]	.25** [.08, .41]																											
7	rVS	0.10	0.14	-.02 [-.20, .15]	.03 [-.15, .20]	.54** [.40, .66]	.42** [.26, .55]	.30** [.13, .46]	.74** [.65, .81]																										
**ROIs pmod empathy**																																			
8	dmPFC	0.05	0.09	.06 [-.12, .23]	.02 [-.15, .20]	.82** [.75, .87]	.49** [.34, .61]	.24** [.07, .40]	.51** [.37, .63]	.51** [.37, .63]																									
9	lAI	0.03	0.10	-.02 [-.20, .16]	-.02 [-.20, .16]	.51** [.36, .63]	.78** [.70, .84]	.25** [.08, .41]	.45** [.29, .58]	.36** [.20, .51]	.63** [.50, .72]																								
10	rAI	0.03	0.08	-.08 [-.25, .10]	-.04 [-.22, .14]	.35** [.19, .50]	.31** [.13, .46]	.83** [.76, .88]	.22* [.05, .38]	.20* [.03, .37]	.35** [.19, .50]	.35** [.18, .50]																							
**ROIs pmod compassion**																																			
11	lVS	0.06	0.10	-.04 [-.21, .14]	-.05 [-.22, .13]	.43** [.28, .57]	.37** [.21, .52]	.10 [-.08, .27]	.62** [.50, .72]	.54** [.40, .66]	.46** [.30, .59]	.44** [.29, .57]	.09 [-.09, .27]																						
12	rVS	0.05	0.08	-.05 [-.22, .13]	-.04 [-.22, .14]	.43** [.27, .56]	.34** [.17, .49]	.14 [-.04, .31]	.54** [.40, .65]	.68** [.57, .76]	.46** [.31, .59]	.37** [.21, .52]	.09 [-.09, .27]	.84** [.78, .89]																					
**IRI self-report**																																			
13	Personal distress	10.8	3.30	-.05 [-.23, .13]	.05 [-.13, .22]	.13 [-.05, .30]	.23* [.05, .39]	.17 [-.01, .33]	.03 [-.14, .21]	.07 [-.11, .25]	.14 [-.04, .31]	.27** [.09, .42]	.13 [-.05, .30]	.10 [-.08, .27]	.18 [-.00, .34]																				
14	Empathic concern	14	2.91	.18 [-.00, .34]	.36** [.19, .50]	.01 [-.17, .18]	.14 [-.04, .31]	.02 [-.15, .20]	.15 [-.03, .32]	.14 [-.04, .31]	.02 [-.16, .19]	.11 [-.07, .28]	-.02 [-.20, .16]	.13 [-.05, .30]	.18 [-.00, .34]	.21* [.03, .37]																			
**EMA everyday**																																			
15	Compassion	57.1	19.8	.33** [.16, .48]	.40** [.24, .54]	-.11 [-.28, .07]	.07 [-.11, .25]	-.06 [-.24, .12]	-.11 [-.28, .07]	-.07 [-.25, .10]	-.13 [-.30, .05]	.12 [-.06, .29]	-.03 [-.20, .15]	.01 [-.17, .19]	.01 [-.17, .19]	.28** [.10, .43]	.42** [.26, .55]																		
16	Distance/ coldness	17.7	13	-.22* [-.38, -.04]	-.21* [-.37, -.03]	.04 [-.14, .21]	.01 [-.17, .19]	.04 [-.14, .22]	.10 [-.08, .27]	.05 [-.13, .23]	.11 [-.07, .28]	.13 [-.04, .31]	.11 [-.07, .28]	.01 [-.17, .19]	.05 [-.13, .23]	.13 [-.05, .30]	-.20* [-.36, -.02]	-.12 [-.29, .06]																	
**EmpaToM behavioral**																																			
17	ToM performance	0.00	0.68	-.02 [-.20, .16]	-.13 [-.30, .05]	.14 [-.03, .31]	-.00 [-.18, .17]	-.14 [-.31, .04]	-.09 [-.27, .09]	-.04 [-.21, .14]	.13 [-.05, .30]	.03 [-.15, .20]	-.16 [-.33, .02]	.07 [-.11, .24]	.06 [-.12, .23]	-.01 [-.18, .17]	-.09 [-.26, .09]	-.05 [-.23, .13]	-.00 [-.18, .17]																
**ROIs ToM contrast questions**																																			
18	mPFC	0.21	0.20	-.01 [-.19, .17]	-.09 [-.26, .09]	.19* [.01, .36]	-.06 [-.23, .12]	.10 [-.08, .27]	.14 [-.04, .31]	.14 [-.04, .31]	.14 [-.03, .31]	.01 [-.17, .19]	.04 [-.14, .22]	.16 [-.02, .33]	.13 [-.05, .30]	.04 [-.13, .22]	.12 [-.06, .29]	.03 [-.15, .20]	.07 [-.11, .25]	.11 [-.07, .28]															
19	PCC	0.31	0.30	-.11 [-.28, .07]	-.04 [-.21, .14]	.06 [-.12, .24]	.00 [-.17, .18]	-.04 [-.21, .14]	.02 [-.16, .19]	.03 [-.14, .21]	.07 [-.10, .25]	.06 [-.12, .24]	-.02 [-.20, .15]	.03 [-.14, .21]	.04 [-.14, .22]	-.00 [-.18, .17]	-.03 [-.21, .15]	-.10 [-.27, .08]	-.03 [-.21, .15]	.13 [-.05, .30]	.31** [.14, .47]														

20	lTP	0.26	0.21	-.09 [-.26, .09]	-.01 [-.19, .17]	.13 [-.05, .30]	-.06 [-.23, .12]	.10 [-.08, .27]	.07 [-.11, .24]	-.01 [-.19, .16]	.13 [-.05, .30]	.04 [-.14, .22]	.14 [-.04, .31]	.11 [-.06, .29]	.07 [-.11, .24]	.13 [-.04, .30]	.06 [-.12, .23]	.02 [-.15, .20]	.09 [-.09, .26]	.13 [-.05, .30]	.38** [.22, .52]	.28** [.11, .43]													
	Variable	*M*	*SD*	1	2	3	4	5	6	7	8	9	10	11	12	13	14	15	16	17	18	19	20	21	22	23	24	25	26	27	28	29	30	31	32
21	rTP	0.36	0.29	.02 [-.16, .19]	-.05 [-.23, .13]	.09 [-.09, .26]	.22* [.04, .38]	-.08 [-.25, .10]	.10 [-.08, .28]	.01 [-.17, .18]	.14 [-.04, .31]	.29** [.12, .44]	-.09 [-.26, .09]	.11 [-.07, .28]	.04 [-.14, .22]	.07 [-.11, .25]	-.12 [-.29, .06]	.12 [-.06, .29]	.01 [-.16, .19]	.28** [.11, .43]	.18* [.00, .35]	.26** [.08, .41]	.24** [.07, .40]												
22	lTPJ	0.29	0.24	-.09 [-.27, .09]	-.12 [-.29, .06]	.02 [-.16, .20]	.06 [-.12, .24]	-.12 [-.29, .06]	.23* [.05, .39]	.13 [-.05, .30]	.12 [-.06, .30]	.20* [.02, .36]	.03 [-.15, .20]	.20* [.03, .37]	.14 [-.04, .31]	-.06 [-.24, .12]	.07 [-.11, .24]	-.01 [-.19, .17]	.03 [-.15, .21]	.08 [-.10, .26]	.34** [.17, .48]	.34** [.17, .48]	.30** [.12, .45]	.25** [.08, .41]											
23	rTPJ	0.12	0.20	-.02 [-.20, .15]	.07 [-.11, .25]	-.09 [-.27, .09]	.01 [-.17, .18]	-.04 [-.22, .13]	.13 [-.05, .30]	.02 [-.16, .19]	-.07 [-.24, .11]	.06 [-.12, .24]	-.09 [-.27, .09]	.11 [-.07, .28]	-.01 [-.19, .17]	.14 [-.04, .31]	.05 [-.13, .23]	.10 [-.08, .28]	.10 [-.08, .27]	-.01 [-.18, .17]	.19* [.02, .36]	.33** [.17, .48]	.21* [.03, .37]	.33** [.16, .48]	.28** [.11, .44]										
**ROIs ToM contrast videos**																																			
24	mPFC	0.08	0.11	.15 [-.03, .32]	.11 [-.07, .28]	.11 [-.07, .28]	.02 [-.16, .20]	.03 [-.15, .21]	.15 [-.03, .32]	.05 [-.13, .22]	.05 [-.13, .22]	-.07 [-.24, .11]	-.06 [-.24, .12]	-.02 [-.20, .16]	-.04 [-.22, .14]	-.12 [-.29, .06]	.10 [-.08, .27]	.08 [-.10, .25]	.15 [-.03, .32]	-.02 [-.20, .16]	.44** [.28, .57]	.13 [-.05, .30]	.08 [-.10, .25]	.07 [-.10, .25]	-.03 [-.21, .15]	.16 [-.01, .33]									
25	PCC	0.12	0.17	-.09 [-.26, .09]	-.02 [-.20, .16]	.01 [-.17, .19]	.01 [-.16, .19]	.03 [-.15, .21]	-.05 [-.22, .13]	-.00 [-.18, .17]	.02 [-.15, .20]	-.03 [-.20, .15]	.00 [-.18, .18]	-.04 [-.22, .14]	-.03 [-.21, .15]	-.21* [-.37, -.03]	-.07 [-.25, .10]	-.10 [-.28, .08]	-.13 [-.30, .05]	.01 [-.17, .19]	.04 [-.14, .22]	.51** [.36, .63]	-.04 [-.21, .14]	.09 [-.09, .26]	.09 [-.09, .26]	.13 [-.05, .30]	.30** [.13, .46]								
26	lTP	0.12	0.15	-.04 [-.21, .14]	-.01 [-.18, .17]	.08 [-.10, .25]	-.01 [-.18, .17]	.11 [-.07, .28]	-.06 [-.23, .12]	-.12 [-.29, .06]	.13 [-.05, .30]	.01 [-.17, .18]	.12 [-.06, .29]	-.12 [-.29, .06]	-.13 [-.30, .05]	.06 [-.12, .23]	-.08 [-.25, .10]	.07 [-.11, .24]	.11 [-.07, .28]	-.01 [-.18, .17]	.12 [-.06, .29]	.08 [-.10, .25]	.36** [.20, .51]	.07 [-.11, .24]	.06 [-.12, .24]	.07 [-.10, .25]	.31** [.14, .46]	.31** [.14, .46]							
27	rTP	0.12	0.18	-.04 [-.21, .14]	-.12 [-.29, .06]	.05 [-.13, .22]	.14 [-.04, .31]	.10 [-.08, .27]	-.04 [-.22, .14]	-.06 [-.23, .12]	.03 [-.15, .21]	.19* [.01, .35]	.12 [-.06, .29]	-.00 [-.18, .18]	-.04 [-.21, .14]	.01 [-.16, .19]	-.08 [-.25, .10]	.03 [-.15, .21]	-.01 [-.19, .17]	.20* [.02, .36]	-.07 [-.25, .10]	-.05 [-.22, .13]	-.11 [-.28, .07]	.42** [.27, .56]	.00 [-.17, .18]	.00 [-.17, .18]	.05 [-.13, .23]	.16 [-.02, .32]	.11 [-.07, .29]						
28	lTPJ	0.16	0.15	-.02 [-.20, .16]	-.07 [-.24, .11]	.04 [-.14, .21]	.11 [-.07, .28]	.00 [-.18, .18]	.07 [-.11, .25]	.04 [-.14, .22]	.10 [-.08, .27]	.09 [-.09, .26]	.01 [-.17, .19]	.03 [-.15, .21]	.02 [-.16, .20]	-.11 [-.28, .07]	.02 [-.16, .20]	.02 [-.16, .20]	.02 [-.15, .20]	.07 [-.11, .25]	.05 [-.13, .23]	.06 [-.12, .23]	-.05 [-.22, .13]	.05 [-.13, .23]	.32** [.15, .47]	.10 [-.07, .28]	.33** [.16, .48]	.46** [.31, .59]	.24** [.07, .40]	.23* [.06, .39]					
29	rTPJ	0.03	0.14	-.15 [-.32, .02]	-.14 [-.31, .04]	.14 [-.04, .31]	.13 [-.05, .30]	.03 [-.14, .21]	.08 [-.10, .25]	.08 [-.10, .25]	.00 [-.18, .18]	.01 [-.17, .19]	-.07 [-.24, .11]	.09 [-.09, .26]	.08 [-.09, .26]	.16 [-.02, .33]	.02 [-.16, .19]	.06 [-.12, .23]	.12 [-.06, .29]	.01 [-.17, .18]	-.03 [-.21, .14]	-.02 [-.20, .16]	-.02 [-.20, .16]	.06 [-.12, .24]	-.12 [-.29, .06]	.21* [.04, .38]	.17 [-.01, .34]	.26** [.09, .42]	.13 [-.05, .30]	.14 [-.04, .31]	.39** [.23, .53]				
**IRI self-report**																																			
30	Fantasy	13.4	3.27	.16 [-.02, .32]	.24** [.06, .40]	.18 [-.00, .34]	.17 [-.00, .34]	.03 [-.15, .20]	.14 [-.04, .31]	.13 [-.05, .30]	.18* [.01, .35]	.22* [.05, .39]	.11 [-.07, .28]	.24** [.07, .40]	.22* [.04, .38]	.22* [.05, .38]	.47** [.32, .60]	.33** [.17, .48]	-.02 [-.20, .15]	.04 [-.14, .21]	.09 [-.09, .26]	.07 [-.11, .24]	.20* [.03, .37]	.02 [-.16, .20]	.16 [-.02, .33]	.15 [-.02, .32]	.02 [-.16, .20]	-.11 [-.29, .06]	-.09 [-.26, .09]	-.01 [-.19, .16]	-.01 [-.18, .17]	-.01 [-.19, .17]			
31	Perspective taking	14.2	2.64	.14 [-.04, .31]	.14 [-.04, .31]	-.05 [-.23, .13]	.04 [-.14, .22]	.01 [-.17, .19]	.11 [-.07, .28]	.06 [-.12, .24]	-.05 [-.22, .13]	.01 [-.16, .19]	-.05 [-.23, .13]	.03 [-.15, .21]	.07 [-.11, .24]	-.09 [-.26, .09]	.33** [.16, .48]	.26** [.09, .42]	-.13 [-.30, .04]	-.07 [-.25, .10]	.07 [-.11, .24]	-.05 [-.23, .13]	-.16 [-.33, .02]	-.00 [-.18, .18]	.03 [-.15, .21]	-.14 [-.31, .04]	.08 [-.10, .26]	-.09 [-.26, .09]	-.00 [-.18, .17]	.13 [-.05, .30]	.11 [-.07, .28]	.08 [-.10, .26]	.02 [-.16, .20]		
**EMA everyday**																																			
32	Perspective taking	57.3	19.8	.42** [.27, .56]	.40** [.24, .54]	.07 [-.11, .24]	.12 [-.06, .29]	-.08 [-.25, .10]	-.02 [-.19, .16]	-.06 [-.24, .12]	.07 [-.11, .24]	.16 [-.02, .33]	-.06 [-.24, .12]	.06 [-.12, .24]	.05 [-.13, .22]	.07 [-.10, .25]	.33** [.16, .48]	.57** [.43, .68]	-.06 [-.23, .12]	.01 [-.16, .19]	.17 [-.01, .34]	-.06 [-.24, .12]	.03 [-.15, .21]	.03 [-.15, .20]	.13 [-.05, .30]	.08 [-.10, .25]	.26** [.09, .42]	-.07 [-.25, .11]	.06 [-.12, .23]	.03 [-.14, .21]	.09 [-.09, .26]	-.06 [-.23, .12]	.31** [.13, .46]	.19* [.01, .35]	
33	Own perspective	48.4	16.1	-.14 [-.31, .04]	-.03 [-.21, .15]	-.14 [-.31, .04]	-.10 [-.28, .07]	.13 [-.05, .30]	-.06 [-.23, .12]	-.07 [-.25, .11]	-.13 [-.30, .05]	-.05 [-.22, .13]	.10 [-.08, .27]	-.11 [-.29, .06]	-.14 [-.31, .04]	.08 [-.10, .26]	-.17 [-.34, .01]	-.06 [-.24, .12]	.17 [-.00, .34]	.00 [-.18, .18]	-.01 [-.19, .17]	.06 [-.12, .23]	.09 [-.08, .27]	.02 [-.16, .20]	.06 [-.12, .24]	.13 [-.04, .31]	-.06 [-.24, .12]	-.05 [-.23, .13]	-.09 [-.26, .09]	-.08 [-.25, .10]	-.06 [-.23, .12]	-.09 [-.26, .09]	-.04 [-.21, .14]	-.11 [-.28, .07]	-.22* [-.38, -.05]

Note. M and *SD* are used to represent mean and standard deviation, respectively. Values in square brackets indicate the 95% confidence interval for each correlation. ROIs = Regions of interest, IRI = Interpersonal Reactivity Index, EMA = Ecological Momentary Assessment, r = right, l = left, VS = ventral striatum, AI = anterior insula, dmPFC = dorsomedial prefrontal cortex, TP = temporal pole, TPJ = temporoparietal junction, PCC = posterior cingulate cortex.

* indicates $p < .05$. ** indicates $p < .001$

Supplementary Table S 8

Means, standard deviations and correlations of the level 1 variables (EMA items or scales) from the multilevel model predicting EMA perspective-taking by mPFC activation in the ToM contrast controlling for situational characteristics.

	M	*SD*	1	2	3	4	5	6
1. Positive content	73.27	25.36						
2. Negative content	28.37	28.89	-.38**					
3. Self as topic	47.92	32.36	.17**	.12**				
4. Other as topic	68.95	28.69	.20**	.15**	.11**			
5. Positive affect	54.84	20.09	.19**	-.07**	.08**	.10**		
6. Negative affect	8.30	12.60	-.32**	.31**	.10**	-.05**	-.08**	
7. Perspective-taking	56.34	32.12	.18**	.10**	.04*	.34**	.16**	-.05**

Note. M and *SD* are used to represent mean and standard deviation, respectively. EMA = Ecological Momentary Assessment, mPFC = Medial Prefrontal Cortex.
* indicates $p < .05$. ** indicates $p < .01$.

Supplementary Table S 9

Characteristics of fixed effects and variance of random effects from the multilevel model predicting EMA perspective-taking by mPFC activation in the ToM contrast during the video epoch controlling for EMA situational characteristics

	Model component	Estimate	SE	p	σ^2 of random effect
	Intercept	52.80	1.87	< .001***	252.19
ROI	mPFC	44	12.75	< .001***	
Situational characteristic	Positive content	0.13	0.03	< .001***	0.02
	Negative Content	0.11	0.02	< .001***	0.02
	Self as topic	- 0.02	0.02	.425	0.02
	Other as topic	0.23	0.03	< .001***	0.04
	Positive affect	0.11	0.03	< .001***	0.02
	Negative affect	- 0.16	0.06	.005**	0.09

Note. All situational characteristics variables grand mean centered. EMA = Ecological Momentary Assessment, ToM = Theory of Mind, mPFC= Medial Prefrontal Cortex, ROI = Region of Interest, SE = Standard Error, σ^2 = Variance.
* indicates $p < .05$. ** indicates $p < .01$. *** indicates $p < .001$.

Supplementary Table S 10

Contingencies of EMA-assessed momentary social affect and cognition on situational characteristics

Variable	Self as topic	Other as topic	Positive content	Negative content	Positive affect	Negative affect
Compassion	.1*** (.3)	.29*** (.27)	.21*** (.29)	.08** (.29)	.1* (.27)	-.22***(.27)
Distance/ coldness	-.05** (.28)	-.08***(.29)	-.26***(.26)	.18*** (.27)	-.09** (.27)	.75*** (.28)
Perspective-taking	-.01 (.3)	.28*** (.28)	.13*** (.28)	.08** (.26)	.16*** (.24)	-.17** (.26)
Focus on own perspective	.35*** (.27)	-.12*** (.3)	.04 (.27)	.01 (.28)	.1**(.25)	.37*** (.26)

Note. Table entries are unstandardized coefficients from multilevel models predicting social affect and cognition variables from situational characteristics. Numbers in parentheses represent standard deviations of the within – person correlation indicating the amount to which participants differ in these contingencies.
* indicates $p < .05$. ** indicates $p < .01$. *** indicates $p < .001$.

Supplementary Figure S 1

The influence of the topic of the communication on momentary social affect differs as a function of social affect network activation

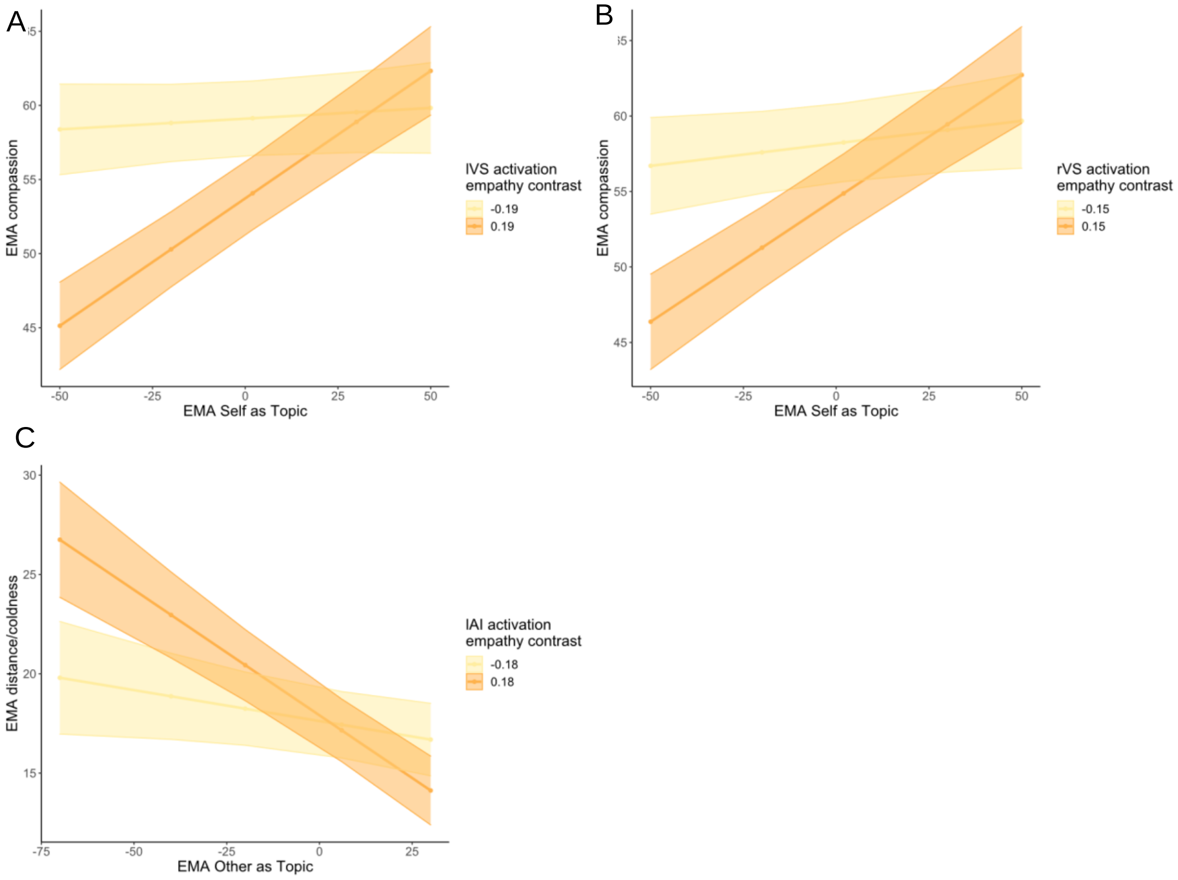

Note. Interaction plots for EMA Self as topic and left (A) and right (B) VS activation in the empathy contrast on EMA compassion as well as EMA Other as topic and left AI activation in the empathy contrast on EMA Distance/coldness (C). Slopes displayed for +/-1 standard deviation from the mean and include standard errors. EMA Self as topic was grand mean centered. EMA = Ecological Momentary Assessment, l = left, r = right, VS = Ventral Striatum, AI = Anterior Insula.

Supplementary Figure S 2

The influence of momentary affect on momentary social cognition differs as a function of social cognition network activation.

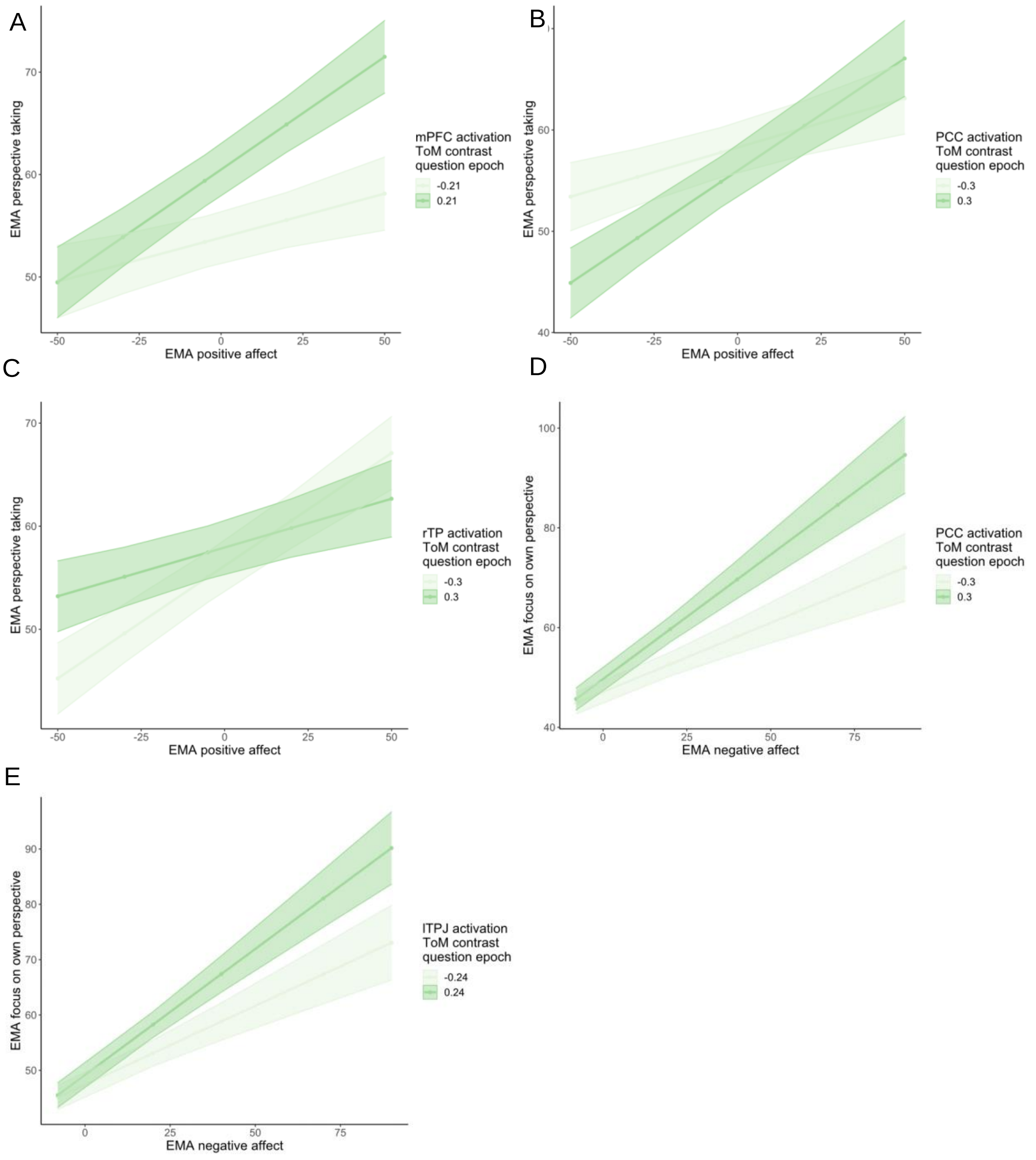

Interaction plots for EMA positive affect and mPFC (A), PCC (B) and right TP (C) activation in the ToM contrast on EMA perspective-taking as well as EMA negative affect and PCC (D) and lTPJ (E) activation in the ToM contrast on EMA focus on the own perspective. Slopes displayed for +/-1 standard deviation from the mean and include standard errors. Predictor variables were grand mean centered. EMA = Ecological Momentary

Assessment, ToM = Theory of Mind, mPFC = Medial Prefrontal Cortex, PCC = Posterior Cingulate Cortex, r = right, TP = Temporal Pole, TPJ = Temporoparietal Junction.

Supplementary Figure S 3

The influence of the topic of the communication on momentary social cognition differs as a function of social cognition network activation.

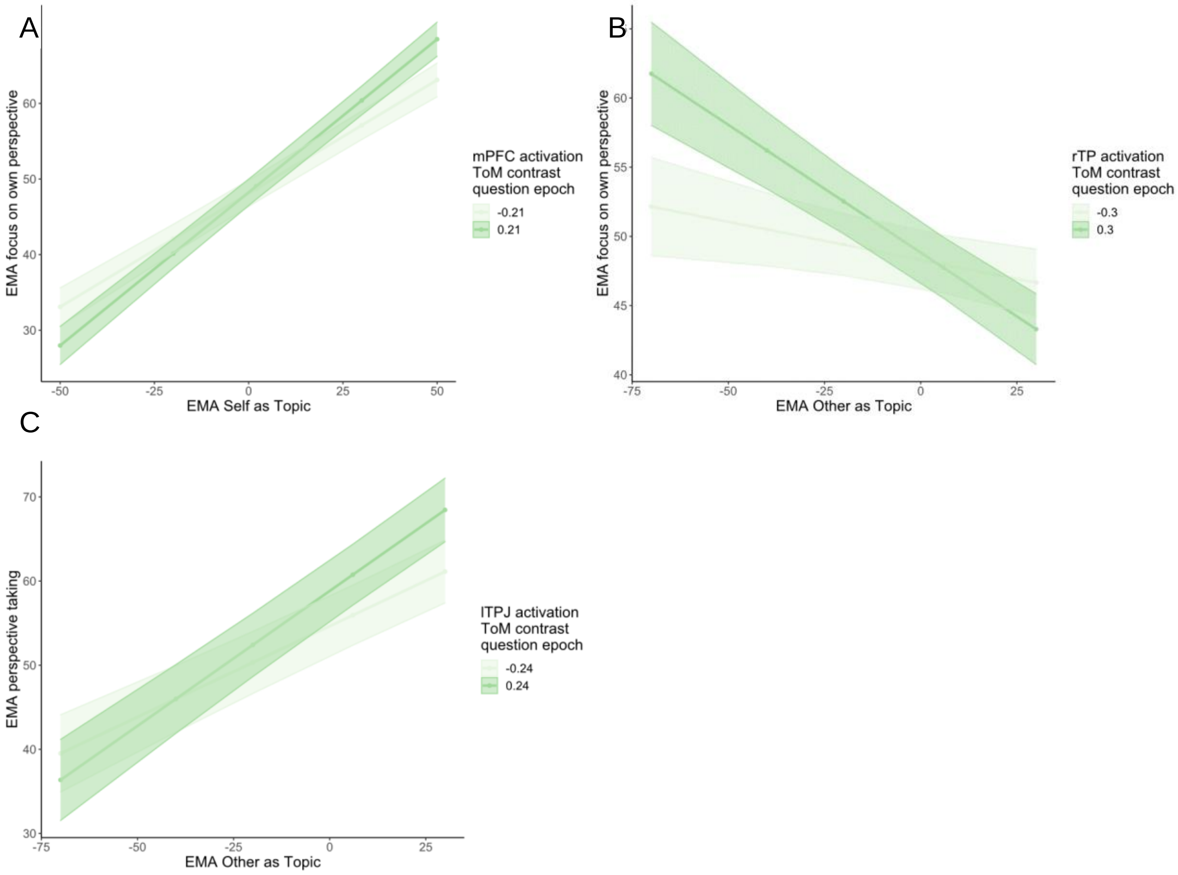

Interaction plots for EMA self as topic and mPFC activation in the ToM contrast (A) and EMA other as topic and right TP activation in the ToM contrast (B) on EMA focus on own perspective, as well as EMA other as topic and left TPJ activation in the ToM contrast (C) on EMA perspective-taking. Slopes displayed for +/-1 standard deviation from the mean and include standard errors. EMA self as topic and other as topic were grand mean centered. EMA = Ecological Momentary Assessment, ToM = Theory of Mind, mPFC = Medial Prefrontal Cortex, r = right, TP = Temporal Pole, TPJ = Temporoparietal Junction.

Supplementary Figure S 4

Intercorrelations of study variables parsed by measurement type — social affect (yellow) / social cognition (green)

EmpaToM behavioral

		1	2	3
empathy	1			
compassion	2	.62		
ToM performance	3	-.02	-.13	

IRI self-report

		1	2		4
personal distress	1				
empathic concern	2	.21			
		.22	.47		
perspective taking	4	-.09	.33	.02	

EMA everyday

		1	2	3	4
compassion	1				
distance/coldness	2	-.12			
**EMA everyday**					
perspective taking	3	.57	-.06		
own perspective	4	-.06	.17	-.22	

EmpaToM neural

			1	2	3	4	5	6	7	8	9	10	11	12	13	14	15	16	17	18	19	20	21	22
empathy	dmPFC	1																						
contrast	lAI	2	.58																					
	rAI	3	.37	.36																				
	VS	4	.53	.55	.25																			
	rVS	5	.54	.42	.30	.74																		
pmod	dmPFC	6	.82	.49	.24	.51	.51																	
emp	lAI	7	.51	.78	.25	.45	.36	.63																
	rAI	8	.35	.31	.83	.22	.20	.35	.35															
pmod	lVS	9	.43	.37	.10	.62	.54	.46	.44	.09														
comp	rVS	10	.43	.34	.14	.54	.68	.46	.37	.09	.84													
questions	mPFC	11	.19	-.06	.10	.14	.14	.14	.01	.04	.16	.13												
	PCC	12	.06	.00	-.04	.02	.03	.07	.06	-.02	.03	.04	.31											
	lTP	13	.13	-.06	.10	.07	-.01	.13	.04	.14	.11	.07	.38	.28										
	rTP	14	.09	.22	-.08	.10	.01	.14	.29	-.09	.11	.04	.18	.26	.24									
	lTPJ	15	.02	.06	-.12	.23	.13	.12	.20	.03	.20	.14	.34	.34	.30	.25								
	rTPJ	16	-.09	.01	-.04	.13	.02	-.07	.06	-.09	.11	-.01	.19	.33	.21	.33	.28							
videos	mPFC	17	.11	.02	.03	.15	.05	.05	-.07	-.06	-.02	-.04	.44	.13	.08	.07	-.03	.16						
	PCC	18	.01	.01	.03	-.05	.00	.02	-.03	.00	-.04	-.03	.04	.51	-.04	.09	.09	.13	.30					
	lTP	19	.08	-.01	.11	-.06	-.12	.13	.01	.12	-.12	-.13	.12	.08	.36	.07	.06	.07	.31	.31				
	rTP	20	.05	.14	.10	-.04	-.06	.03	.19	.12	.00	-.04	-.07	-.05	-.11	.42	.00	.00	.05	.16	.11			
	lTPJ	21	.04	.11	.00	.07	.04	.10	.09	.01	.03	.02	.05	.06	-.05	.05	.32	.10	.33	.46	.24	.23		
	rTPJ	22	.14	.13	.03	.08	.08	.00	.01	-.07	.09	.08	-.03	-.02	-.02	.06	-.12	.21	.17	.26	.13	.14	.39	

Columns 1–5: *empathy contrast*; 6–8: *pmod emp*; 9–10: *comp* (neural); 11–16: *questions*; 17–22: *videos* (neural)

Note. Heatmaps of bivariate Pearson's correlations of all person-level variables parsed my measurement type. Alternative presentation of the data in figure 2. Correlations ≤ r = -.18 and ≥ r = .18 are significant (uncorrected p). MFG = middle frontal gyrus, PCG = postcentral gyrus, IFG = inferior frontal gyrus, TPJ = temporoparietal junction, r = right, l = left, VS = ventral striatum, AI = anterior insula, dmPFC = dorsomedial prefrontal cortex, TP = temporal pole, PCC = posterior cingulate cortex, pmod = parametric modulation.
